# Screening and verification of extranuclear genetic markers in green tide algae from the Yellow Sea

**DOI:** 10.1371/journal.pone.0250968

**Published:** 2021-06-01

**Authors:** Chuner Cai, Kai Gu, Hui Zhao, Sophie Steinhagen, Peimin He, Thomas Wichard

**Affiliations:** 1 College of Marine Ecology and Environment, Shanghai Ocean University, Shanghai, China; 2 Institute for Inorganic and Analytical Chemistry, Jena School for Microbial Communication, Friedrich Schiller University Jena, Jena, Germany; 3 Department of Marine Sciences-Tjärnö Marine Laboratory, University of Gothenburg, Strömstad, Sweden; Austrian Federal Research Centre for Forests BFW, AUSTRIA

## Abstract

Over the past decade, *Ulva compressa*, a cosmopolitan green algal species, has been identified as a component of green tides in the Yellow Sea, China. In the present study, we sequenced and annotated the complete chloroplast genome of *U*. *compressa* (alpha-numeric code: RD9023) and focused on the assessment of genome length, homology, gene order and direction, intron size, selection strength, and substitution rate. We compared the chloroplast genome with the mitogenome. The generated phylogenetic tree was analyzed based on single and aligned genes in the chloroplast genome of *Ulva* compared to mitogenome genes to detect evolutionary trends. *U*. *compressa* and *U*. *mutabilis* chloroplast genomes had similar gene queues, with individual genes exhibiting high homology levels. Chloroplast genomes were clustered together in the entire phylogenetic tree and shared several forward/palindromic/tandem repetitions, similar to those in *U*. *prolifera* and *U*. *linza*. However, *U*. *fasciata* and *U*. *ohnoi* were more divergent, especially in sharing complementary/palindromic repetitions. In addition, phylogenetic analyses of the aligned genes from their chloroplast genomes and mitogenomes confirmed the evolutionary trends of the extranuclear genomes. From phylogenetic analysis, we identified the *pet*A chloroplast genes as potential genetic markers that are similar to the *tuf*A marker. Complementary/forward/palindromic interval repetitions were more abundant in chloroplast genomes than in mitogenomes. Interestingly, a few tandem repetitions were significant for some *Ulva* subspecies and relatively more evident in mitochondria than in chloroplasts. Finally, the tandem repetition [GAAATATATAATAATA × 3, abbreviated as TRg)] was identified in the mitogenome of *U*. *compressa* and the conspecific strain *U*. *mutabilis* but not in other algal species of the Yellow Sea. Owing to the high morphological plasticity of *U*. *compressa*, the findings of this study have implications for the rapid non-sequencing detection of this species during the occurrence of green tides in the region.

## Introduction

Green algae extranuclear genomes date back at least one billion years [1] and account for 0.1% of the nuclear genome. They are used to forecast the evolutionary trends of various species [2, 3]. A phylogenetic analysis of chloroplasts from green algae, for example, discovered a new lineage sister to the Sphaeropleales [4]. Trebouxiophyceae species were multiphyletic in the subphylum, and three clusters were included in the core trebouxiophyceans [5, 6]. Furthermore, the Chlorophyceae contain two robustly supported major lineages, including a clade that unites the Oedogoniales, Chaetophorales, and Chaetopeltidales, as well as a clade that unites the Sphaeropleales and Chlamydomonadales, where *Chlamydomonas reinhardtii* contains putative tyrosine kinases that function in phosphotyrosine signal, which most probably appeared before the animal and fungal lineages were diverged [7, 8].

Pedinophyceae and Trebouxiophyceae, on the other hand, are classified based on the sequences of their nuclear and plastid-encoded ribosomal RNA (rRNA) operons [9]. Ulvophyceae evolution was studied using phylogenetic analysis of seven nuclear genes, small subunit nuclear rDNA, and the plastid genes *rbc*L and *atp*B [10], whereas Ulvales evolution was studied using *rbc*L and small subunit nuclear rDNA [11]. Results from these studies revealed a few unusual structural features based on the chloroplast genomes of individual species. For example, although Pseudendoclonium cpDNA features a large inverted repeat, its quadripartite structure is unusual in that it displays an rRNA operon transcribed toward the large single-copy region and a small single-copy region containing 14 genes that are normally found in the large single-copy region [12]. Moreover, *Ulva* spp. share different numbers of introns [13]. Only a few studies have investigated chloroplast genome-based phylogeny within the genus *Ulva*, and no comparisons have been made with their corresponding mitogenomes [13, 14].

Several representatives of the genus *Ulva* exhibit strong morphological plasticity, and respective morphotypes can vary depending on changing environmental factors [15]. This often resulted in errors in the classical morphological criteria for identification [16–18], especially within subspecies. For instance, the species dominating the green tides in the Yellow Sea was first identified as *U*. *prolifera* in 2008 [19–22], and was subsequently amended as a new subspecies, namely, *U*. *prolifera* subsp. *qingdaoensis* [18]. Although internal transcribed spacers (ITS) and *rbc*L gene sequences are widely used in the classification of green tide algae, they cannot be used to distinguish the subspecies from the species complex “*Ulva linza*-*procera-prolifera”* [23–27]. However, the combination of ITS and 5S *r*DNA could be applied to characterize *U*. *prolifera* from *U*. *linza* [24, 26–35]. Moreover, in some cases, the *tuf*A marker gene possessed, e.g. between *U*. *prolifera* and *U*. *linza*, a higher sensitivity than the DNA barcodes ITS and *rbc*L [18, 36, 37]. Nevertheless, there was a lack of systematic screening for suitable barcodes in characterized regions of whole mitochondrial and chloroplast genomes. Genetic markers were developed within the whole nuclear genomes to identify green-tide forming algae, including inter-simple sequence repeats [19], PCR-restriction fragment length polymorphisms [29], and *in situ* hybridization techniques [38]. Although these methods were accurate, they were time-consuming and entailed high costs.

In the present study, we aimed to (i) identify, sequence, and annotate the complete chloroplast genome of *U*. *compressa*, (ii) perform comparative phylogenetic analyses based on single and aligned genes in the chloroplast genome within the genus *Ulva* for comparison with those from mitogenomes, to determine evolutionary trends, and finally (iii) systematically screen for characteristic regions among the whole mitogenomes and chloroplast genomes of the genus *Ulva* for DNA barcoding purposes. To this end, we sequenced and annotated the complete chloroplast genome of *U*. *compressa* (alpha-numeric code: RD9023), a constitutive species of the green tide in the Yellow Sea, China. We hypothesized that specific nucleotide sequences across the whole mitochondrial and chloroplast genomes can distinguish *U*. *compressa* from other species, considering a rapid non-sequencing approach, and subsequently be used for the identification of evolutionary trends. Phylogenetic analyses were performed and a comparative analysis was carried out for available chloroplast and mitochondrial genomes. Finally, systematic screening was performed for unique barcodes within characterized regions of mitogenomes and chloroplast genomes using algal strains collected from China (the Yellow Sea) and Europe (the North Sea and Baltic Sea).

## Materials and methods

### Sampling and species identification

The specimens of *U*. *compressa* used in this study to unravel the chloroplast genome were collected from the southern Yellow Sea near the Rudong Sea area in Jiangsu Province [39]. Healthy individual algae were selected and washed several times with sterile seawater autoclaved at 121°C for 15 min. Following the removal of any surface attachments, they were cultivated in VSE medium [40] at 24°C under a 12 h photoperiod and photon flux of 130–160 μmol·m^-2^ s^-1^ before being further characterised via ITS [41] and *tuf*A molecular barcoding. Additionally, *Ulva* samples collected from either China (Yellow Sea) or Europe (North and Baltic Sea) were assessed for further comparison (e.g., using phylogenetic trees and barcodes) [16]. No endangered or protected species were targeted at the sampling sites mentioned, and no specific permissions were required for these locations and activities.

### Sequencing and annotation

Several published and peer-reviewed chloroplast genomes (NC_035823 [13], NC_029040 [42], KP720616 [43], and NC_030312 [44]) from representatives of the genus *Ulva* were used as a reference for primer design to sequence the chloroplast genome of *U*. *compressa*. After the pre-test, selected primers were divided into 15 pairs (S1 Table in S1 File) for PCR amplification under the following conditions: pre-denaturation at 94°C for 1 min, 35 cycles of amplification each at 94°C for 30 s, 55°C for 30 s, and 68°C for 8 min, followed by a final extension at 68°C for 15 min. The PCR mixture contained 0.5 μL of DNA polymerase (5 U·μL^−1^), 5 μL of buffer (Mg^2+^ Plus; 10×), 8 μL of dNTP mixture (2.5 mM each), 0.2–1.0 μM (final concentration) of forward and reverse primers, and purified chloroplast DNA (<1 μg). RNase-free water was added to increase the final reaction volume to 50 μL. Takara *LA Taq* polymerase (Takara Biomedical Technology Co., Ltd, Beijing, China) was used for fragments longer than 4,000 bp, whereas Takara *Ex Taq* polymerase (Takara Biomedical Technology Co., Ltd, Beijing, China) used for shorter fragments. PCR products of long segments were fragmented using ultrasound, connected to the PMD19-T vector, and transformed into *E*. *coli* DH5α (Sangon Biotech Co., Ltd., Shanghai, China). Positive clones were selected for shotgun sequencing using the ABI3730 sequencer (Applied Biosystems Co., US). Raw sequence reads were trimmed using cross_match v1.09 and assembled using phred v1.09 [45].

The *U*. *compressa* chloroplast genome was annotated with CpGAVAS in which the cutoff for the E-values of BLASTN and BLASTX was 1·e^−10^ [46]. Meanwhile, transfer RNA (*t*RNA) genes were identified using *t*RNAscan-SE [42] and ARAGORN [47]. Inverted repeat (IR) sequences were predicted using Gepard [48], and the circular chloroplast genome map of *U*. *compressa* was drawn using OrganellarGenomeDRAW [49]. Furthermore, GC content was determined using the Compseq program provided by EMBOSS [50]. Final genome assembly and annotation results were deposited in GenBank (accession number: KX595275).

Simple sequence repeats (SSRs) were detected using the MISA Perl script available at http://pgrc.ipk-gatersleben.de/misa/, at the following thresholds: eight repeat units for mononucleotide SSRs, four repeat units for di- and tri-nucleotide repeat SSRs, and three repeat units for tetra-, penta-, and hexa-nucleotide repeat SSRs. Tandem repeats were analyzed using the Tandem Repeats Finder program [51] by setting two parameters for matches and seven for indels and mismatches, in which 500 and 50 were set as maximum period size and minimum alignment score. After manual verification, irrelevant results were removed. The REPuter program [52] was employed to identify different IR sequences (forwarding, reverse, complementary, and palindromic) in *U*. *compressa*. We used 30 bp as the minimal repeat size, and the homology between repeat units was above 90%.

### Comparative analysis of chloroplast genomes

Reference chloroplast genomes of *U*. *mutabilis* (txid498180), *U*. *flexuosa* (NC_035823), *U*. *linza* (NC_030312), *U*. *prolifera* (NC_036137), *U*. *ohnoi* (AP018696), *U*. *fasciata* (NC_029040), and *Ulva* sp. UNA00071828 (KP720616) were subjoined into a comparative genome analysis of *U*. *compressa* that assessed genome length and homology, gene order and direction, intron size, selection force, and substitution rate. The selection force and substitution rate assessment of 71 protein-coding genes (PCGs) extracted from *Ulva* species using the *KaKs*_Calculator Toolbox 2.0 [53] were performed using γ-NG methods and the standard genetic code [54]. *Ulva compressa* was considered as control during this assessment. In particular, *U*. *compressa* was compared with the conspecific *U*. *mutabilis* Føyn that was developed into a model organism [55, 56].

### Phylogenetic tree analysis

Species with different gene combinations were subjected to phylogenetic analyses using either a group of or single genes. Firstly, the evolutionary status of seven species within genus *Ulva* (*U*. *compressa*, *U*. *mutabilis*, *U*. *flexuosa*, *U*. *linza*, *U*. *prolifera*, *U*. *ohnoi*, and *U*. *fasciata*) was deciphered from the aligned genes of the chloroplast genomes, including 71 PCGs. To compare the results of this analysis, the mitochondrial genome-related phylogenetic analysis was subsequently performed using similar types of mitogenomes from the NCBI database. For each species, 29 aligned PCGs were used. *Pseudendoclonium akinetum* was used as an outgroup in each of these phylogenetic analyses.

Additionally, a phylogenetic tree showing PCG, *r*RNA (*rrn*5, *rrn*16, and *rrn*23), and the alignment of all *t*RNAs from the seven chloroplast genomes mentioned above was constructed separately.

Finally, the individual genes whose phylogenetic trees were in line with that of the whole chloroplast genome were selected for *in silico* PCR using designed primers. After comparison, two coding genes, namely *pet*A and *tuf*A, whose phylogenetic trees were in line with that of the whole chloroplast genome, were selected to classify the samples obtained from the Yellow Sea in China (S2 Table in S1 File).

Alignments were performed using ClustalW [57], manually examined, and adjusted. Each of the phylogenetic trees was analyzed based on maximum likelihood method using the best model tested in MEGA X [58]. The strength of support of branches in the phylogenetic tree was assessed by bootstrap testing with 1,000 replications.

### Non-coding sequence analysis

Complement, forward, and palindromic matches from either chloroplast genomes (over 20 bp) or mitogenomes (over 30 bp) were compared separately to identify the consensus sequences among seven species in the genus *Ulva* (*U*. *compressa*, *U*. *mutabilis*, *U*. *flexuosa*, *U*. *linza*, *U*. *prolifera*, *U*. *ohnoi*, and *U*. *fasciata*). Furthermore, unique tandem repeats in these genomes were revealed.

Twelve pairs of primers were designed across both sides of selected tandem repeats in the chloroplast (three pairs) and mitochondrial (nine pairs) DNA from *U*. *compressa*. These were used to amplify DNA from four constitutive green tide species in the Yellow Sea (S3 Table in S1 File). The PCR products were tested by agarose gel electrophoresis and sequenced to identify unique bands.

Two primer pairs (m-*cox*3-*trn*R and m-*trn*G-*trn*Y) were further used to test 22 strains of green tide samples from the Yellow Sea (S4 Table in S1 File). The subsequent analysis focused on the exclusive pair of primers (m-*trn*G-*trn*Y) that was tested on *U*. *mutabilis* (wild-type and “slender”–a naturally occurring developmental mutant), conspecific with *U*. *compressa* as well as on 27 *Ulva* samples from the North and Baltic Seas (Europe) (S5 Table in S1 File) and *U*. *ohnoi* (origin: Mediterranean Sea).

## Results

### Sequencing and annotation of the chloroplast genome

**Fig 1:**
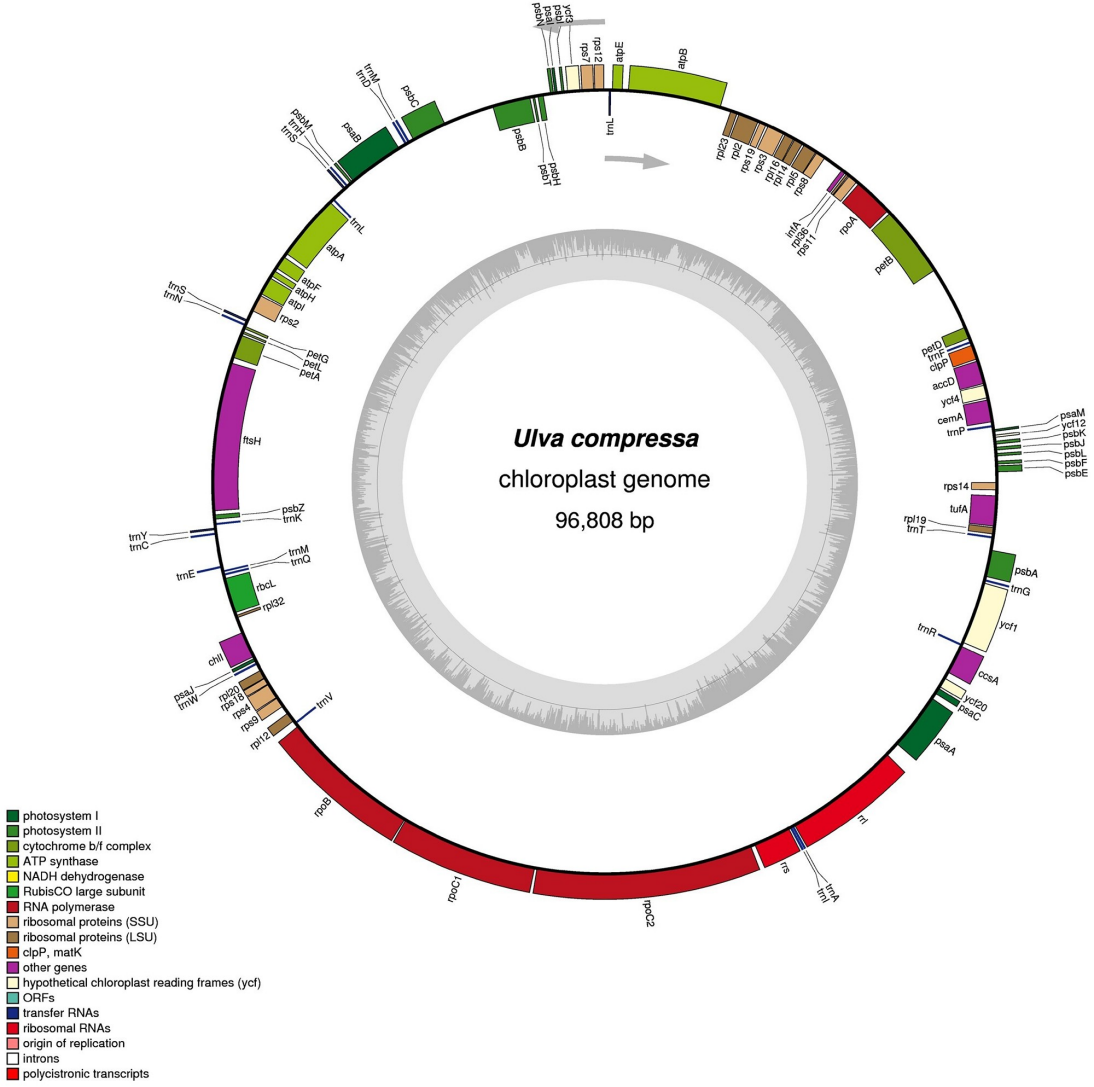
Schematic representation of the *U*. *compressa* chloroplast genome using OGDRAW. The predicted genes are shown, with the colors representing functional classifications at the bottom left. The genes outside the circle were transcribed counterclockwise. The inner-circle shows the GC content.

Sequencing analysis revealed the complete chloroplast genome of *U*. *compressa* (accession number: KX595275) with 96,808 bp and no IR region. The chloroplast genome clustered with peer-reviewed sequences (downloaded from GenBank) previously identified as *U*. *compressa* (S1 Fig in S1 File). One hundred genes were identified, including 71 PCGs, 26 *t*RNA genes, and three *r*RNA genes (S6 Table in S1 File). The general structure and location of these genes was typical for a chloroplast genome in *Ulva* (Fig 1). Six introns were predicted, one each in *pet*D, *pet*B, *atp*B, and *atp*A, and two in *rrn*23. Overall, 74.0% of the genome was composed of genes that encode proteins. The overall GC content of the genome was 26.2%, and 26.5% was allocated to protein-coding regions. Within the latter regions, the GC content for the first, second, and third positions of codons was 34.4%, 31.2%, and 13.8%, respectively.

A total of 235 SSRs were identified in the *U*. *compressa* chloroplast genome. Among these, the majority consisted of mono- and di-nucleotide repeats, which were detected 148 and 42 times, respectively. All of the mononucleotide repeats were A/T repeats. Similarly, the majority of dinucleotide repeats (92.9 percent) were AT/AT repeats. Tri-(19), tetra-(13), penta-(3), and hexa-(8) nucleotide repeats were detected at a lower frequency, with 24 localized in the intergenic regions, 17 in the coding regions, and others stretched across both the intergenic and coding regions (S7 Table in S1 File).

Eighteen tandem repeats longer than 20 bp were identified with a similarity > 90%. Thirteen tandem repeats were located in the intergenic regions, and the remaining within coding regions. Additionally, 10 forward repeats were identified with a size cutoff of 30 bp (S8 Table in S1 File). Among these, the most extended units were 63 bp and were located in the coding regions of *psa*B and *psa*A.

### Comparative analysis of chloroplast genomes

**Fig 2:**
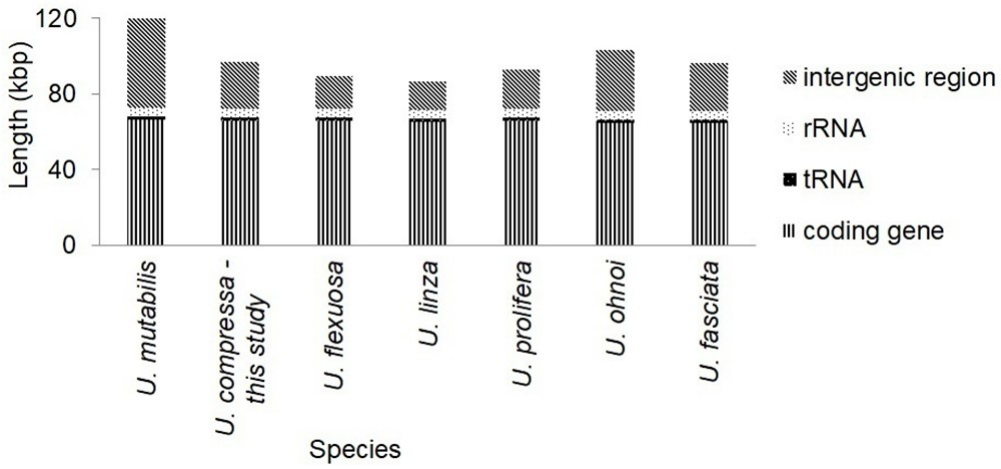
Length of the chloroplast genomes in genus *Ulva*. The total length of intergenic regions varied widely compared to that of the corresponding PCGs, *r*RNAs, and *t*RNAs.

The length of the chloroplast genome from the genus *Ulva* ranged from 86,726 bp (*U*. *linza*) to 119,855 bp (*U*. *mutabilis*) and the number of bases in non-coding regions varied largely compared to that of PCGs, *r*RNAs, and *t*RNAs, which accounted for 60%–80% of the whole genome (Fig 2).

The direction of gene transcription was slightly inconsistent in the case of 7 PCGs and 14 *t*RNAs from the selected 7 species. *Ulva linza* and *U*. *prolifera* had many in common, as did *U*. *ohnoi* and *U*. *fasciata*. However, this was not the case between *U*. *compressa* and *U*. *mutabilis* (S9 Table in S1 File).

All the assessed species, except for *U*. *linza*, contained introns that were spread irregularly within the genus, and a significantly higher number of intron regions were found in *U*. *mutabilis* compared to other *Ulva* species. Notably, 11 introns were included in *U*. *mutabilis*, whereas *U*. *compressa* contained only six. A loss of intronic genes may explain the difference in the number of introns among the species. However, these conclusions were derived from bioinformatic analyses that require further testing to be confirmed.

**Fig 3:**
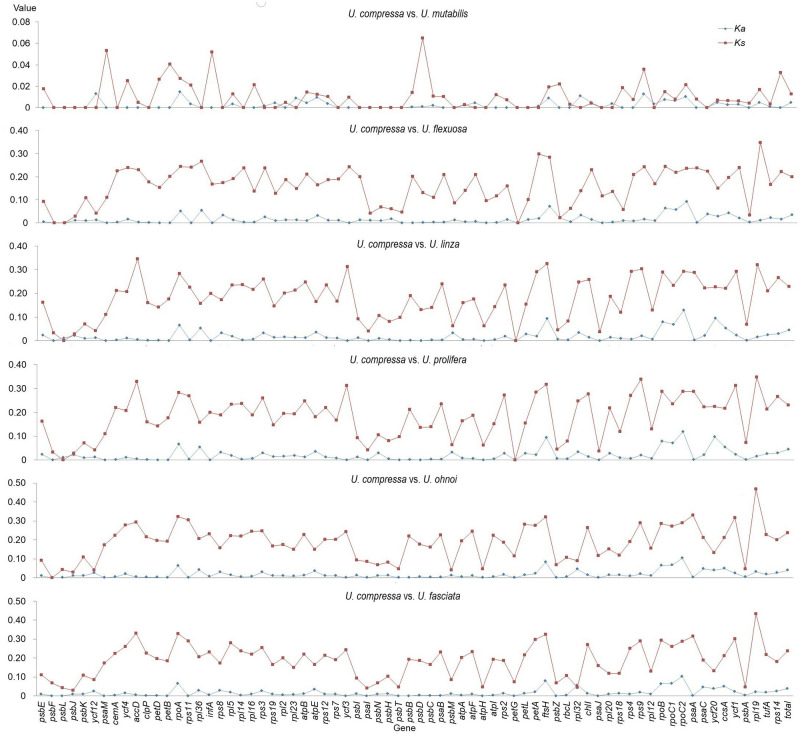
*Ka/Ks* value assessment of 71 PCGs in the chloroplast genomes of seven species. The value was measured using the *KaKs*_Calculator Toolbox 2.0, the γ-NG method, and the standard genetic code. *Ulva compressa* (NCBI #KX595275) was used as a control.

To understand the evolutionary trends of the investigated species, the *Ka/Ks* values of six species were compared against *U*. *compressa* (control) using 71 PCGs (Fig 3). The *Ka/Ks* ratio estimates the balance between neutral mutations, purifying selection)s, and beneficial mutations based on a set of homologous PCGs. Except for *ycf*12, *rps*3, *rps*19, *rpl*23, *atp*F, *pet*A, *rpl*32, *chl*I, *rpl*20, and *rpl*12 in *U*. *mutabilis*, the *Ka* value was always lower than the *Ks* value (i.e., *Ka/Ks* ≤ 1). Moreover, both *Ka* and *Ks* values were distinctly lower (approximately 1/10) in *U*. *mutabilis* compared to the other analyzed species (Fig 3). The average selective constraint in *U*. *mutabilis* (0.38) and other *Ulva* species (0.17–0.20) is purifying selection, according to the *Ka/Ks* ratio. Furthermore, the jagged curve raised several hypotheses for tracing evolutionary changes in these genomes, with *U*. *linza* and *U*. *prolifera*, as well as *U*. *ohnoi* and *U*. *fasciata*, acting nearly identically according to the curve and gene queues.

### Phylogenetic tree based on extranuclear genomes

**Fig 4:**
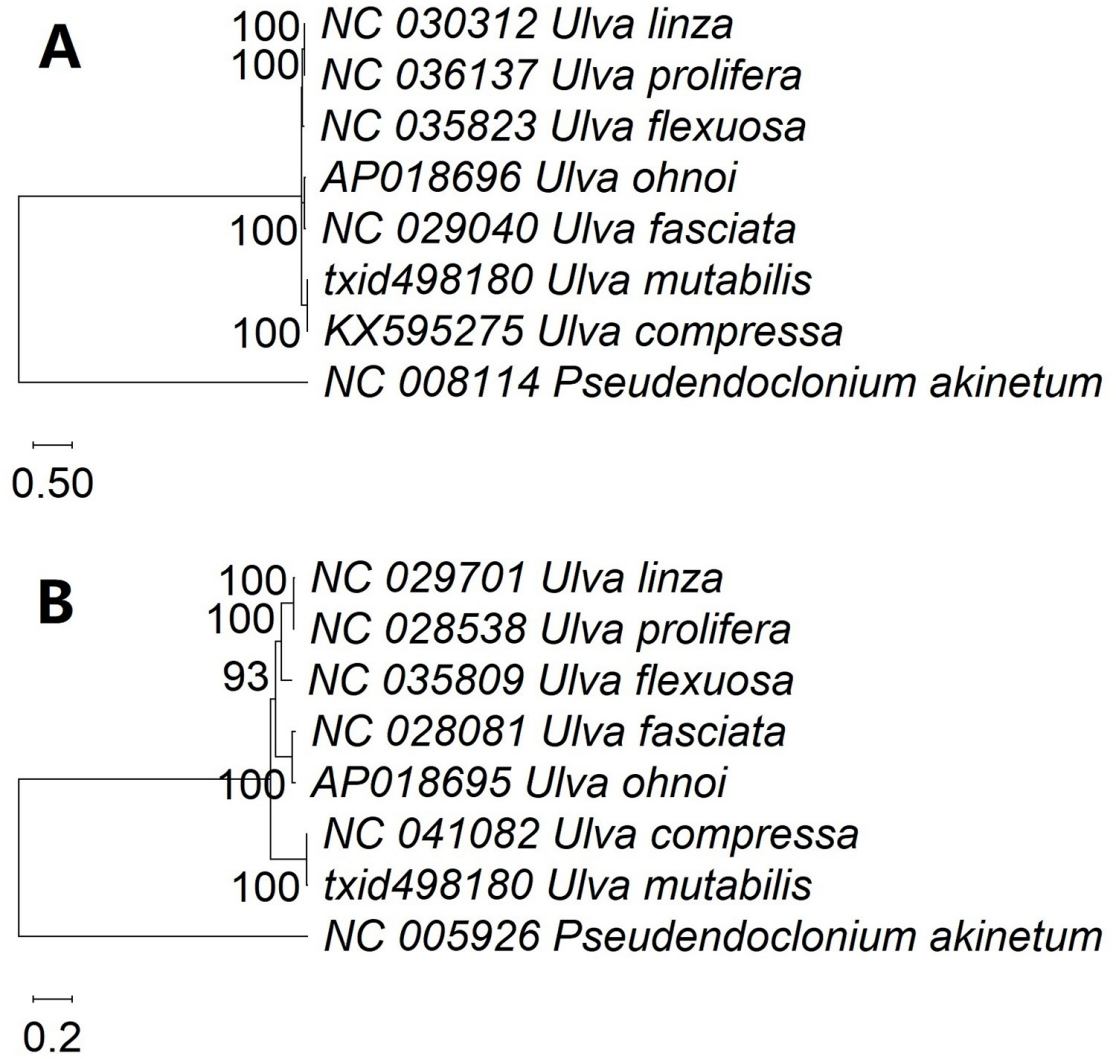
Molecular phylogenetic analysis of *Ulva* species using chloroplast or mitochondrial genomes and the maximum likelihood method based on the General Time Reversible+G+I model (A) and General Time Reversible+G model (B). (A) Molecular phylogenetic analysis with whole aligned coding genes of the *Ulva* chloroplast genomes. The coding genes include *acc*D, *atp*A, *atp*B, *atp*E, *atp*F, *atp*H, *atp*I, *ccs*A, *cem*A, *chl*I, *clp*P, *fts*H, *inf*A, *pet*A, *pet*B, *pet*D, *pet*G, *pet*L, *psa*A, *psa*B, *psa*C, *psa*I, *psa*J, *psa*M, *psb*A, *psb*B, *psb*C, *psb*D, *psb*E, *psb*F, *psb*H, *psb*I, *psb*J, *psb*K, *psb*L, *psb*M, *psb*N, *psb*T, *psb*Z, *rbc*L, *rpl*12, *rpl*14, *rpl*16, *rpl*19, *rp*l2, *rpl*20, *rpl*23, *rpl*32, *rpl*36, *rpl*5, *rpo*A, *rpo*B, *rpo*C1, *rpo*C2, *rps*11, *rps*12, *rps*14, *rps*18, *rps*19, *rps*2, *rps*3, *rps*4, *rps*7, *rps*8, *rps*9, *tuf*A, *ycf*1, *ycf*12, *ycf*20, *ycf*3, and *ycf*4. (B) Molecular phylogenetic analysis with whole aligned coding genes of the *Ulva* mitochondrial genomes. The coding genes include *atp*1, *atp*4, *atp*6, *atp*8, *atp*9, *cob*, *cox*1, *cox*2, *cox*3, *nad*1, *nad*2, *nad*3, *nad*4, *nad*4L, *nad*5, *nad*6, *nad*7, *rpl*14, *rpl*16, *rpl*5, *rps*10, *rps*11, *rps*12, *rps*13, *rps*14, *rps*19, *rps*2, *rps*3, and *rps*4.

We used aligned PCGs from chloroplast genomes to divide the seven *Ulva* species into four groups for the phylogenetic analysis (Fig 4A). The pairs of *Ulva linza* and *Ulva prolifera*, *Ulva ohnoi* and *Ulva fasciata* as well as *Ulva mutabilis* and *Ulva compressa* belonged to the same group, while *U*. *flexuosa* was more closely related to *Ulva linza* and *Ulva prolifera*.

The mitogenome-related phylogenetic analysis of 29 aligned PCGs elucidated the evolutionary status, which was consistent with that of the chloroplast genomes of the seven analzsed species (Fig 4B). The cluster was encountered within all the samples from the same species.

### Identification of *tuf*A and *pet*A markers

A specific type of phylogenetic tree was discovered in a phylogenetic analysis of all PCGs from chloroplast genomes (S2 Fig in S1 File). In this type, each calculation that considered any of the PCGs, including *rbc*L, *rpo*A, *rpo*B, *rpo*C1, *fts*H, *rpo*C2, *psa*A, *psb*I, *ycf*12, *pet*B, *rps*11, *rps*7, *ycf*4, *atp*B, *cem*A, *chl*I, *clp*P, *pet*A, *rpl*5, *tuf*A and *ycf*20, resulted in a similar phylogenetic tree that was also consistent with the tree obtained from whole chloroplast genomes (Fig 4A). But phylogenetic tree from *r*RNAs (S3 Fig in S1 File) and *t*RNAs (S4 Fig in S1 File) were not the same as that from the whole chloroplast genomes.

**Fig 5:**
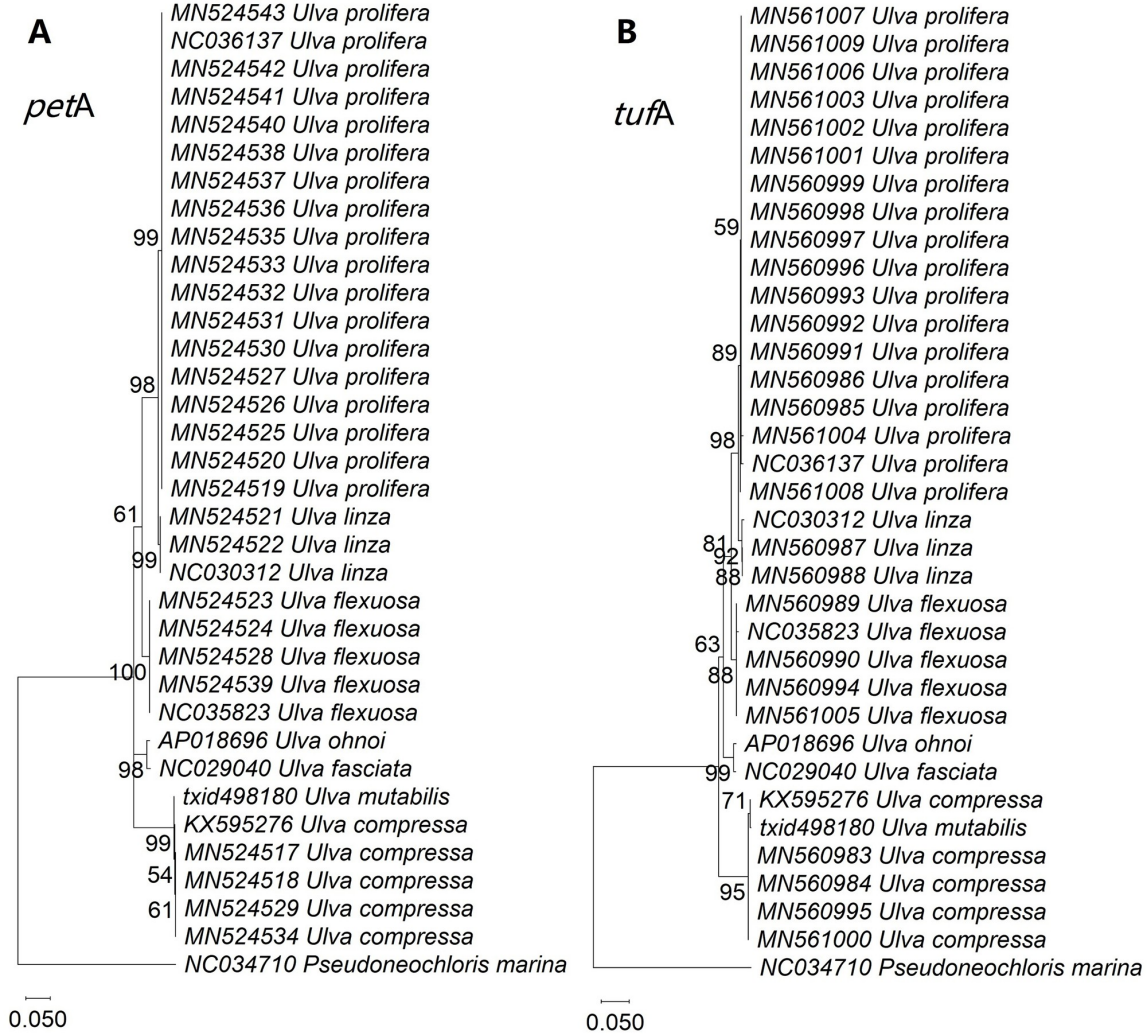
Phylogenetic analysis of *pet*A (A) and *tuf*A (B) from the strains of the green tides in the Yellow Sea, and data from NCBI obtained via the maximum likelihood method in the Tamura 3-parameter+G model. S2 Table in S1 File contains information on the green tide strains in the Yellow Sea.

We aimed to characterize genes from the above special coding gene group, which could potentially function as a suitable barcode for species identification, similar to *tuf*A (1.2k bp). A suitably-sized barcoding marker (e.g., 1.2k ± 0.3k bp) should contain the conserved regions and avoid most non-conserved ones. However, most of the elucidated genes were unsuitable. Some genes were too long (e.g., *rpo*B, *rpo*C1, *rpo*C2) or too short (e.g., *chl*I, *psa*A, *clp*P, *fts*H, *psb*I, *rpl*5, *rps*11, *rps*7, *ycf*12, *ycf*20, *ycf*4) to work as a barcode. Others contained non-conserved regions, e.g., introns, in their genome sequences (e.g., *pet*B, and *atp*B). A few genes caused problems in the design of suitable primers, and the PCR simulation failed (e.g., *cem*A and *rpo*A). Finally, *pet*A sequences allowed us to identify green tide samples from the Yellow Sea, as did the control analysis using *tuf*A (Fig 5).

### Duplication of non-coding sequences in extranuclear genomes

In addition to assessing the PCGs, they were screened for other regions focusing on non-coding sequences among the whole mitogenomes and chloroplast genomes for DNA barcoding purposes. IR sequences such as complementary, forward, or palindromic repeats and tandem repeat sequences were relatively conserved among the non-coding regions. The IR sequences were not as species-specific as the tandem repeats, either in mitochondrial or chloroplast genomes. In some species of *Ulva*, shared IRs were discovered in the chloroplast genomes (S10 Table in S1 File, #1–13), including two complement (#1–2), three forward (#3–5), and eight palindromic matches (#6–13). Six IRs were shared by *U*. *linza* and *U*. *prolifera*, and three by *U*. *compressa* and *U*. *mutabilis*. However, *U*. *linza* and *U*. *prolifera* had only one IR (84 bp) in the mitogenome in common (S10 Table in S1 File, #14).

The tandem repeats appeared to differ among the species significantly but were identical within the subspecies. Forty repeats were found in mitogenomes, with a longer average length (16 bp) than that in chloroplast genomes (11 bp), which only contained six. In mitogenomes, *U*. *compressa* and *U*. *mutabilis* shared nine tandem repeats and four in chloroplast genomes, respectively, whereas *U*. *linza* and *U*. *prolifera* shared eight and two tandem repeats, respectively (S11 Table in S1 File). The longest tandem repeat (35 bp) was found in *Ulva fasciata* (KU182748 and NC 028081), but *U*. *prolifera* (KU161104 and NC 028538) had the highest copy number of an individual tandem repeat (n = 20) (S11 Table in S1 File).

### Non-coding sequence in the extranuclear genome

**Fig 6:**
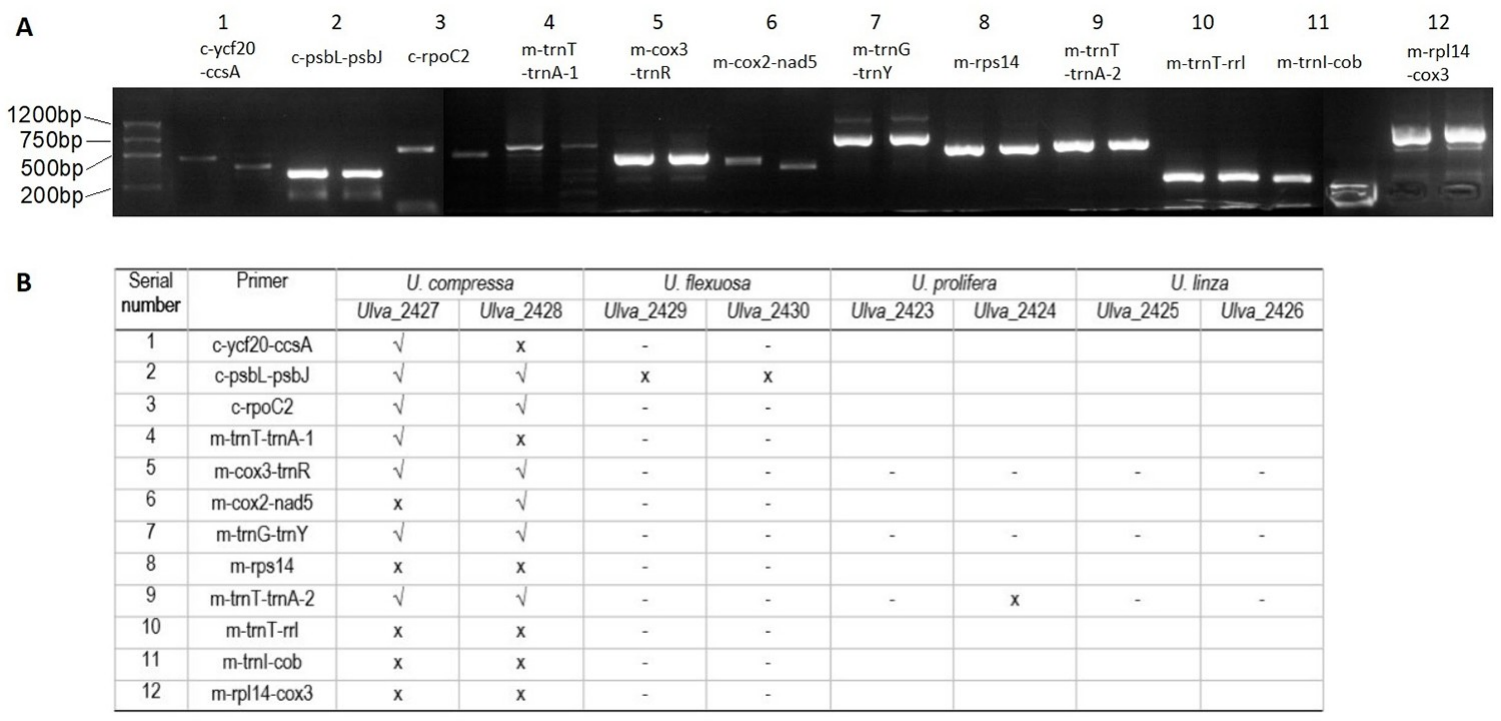
Testing 12 primer pairs on four constitutive green tide species in the Yellow Sea. (A) Electrophoresis results following PCR using *U*. *compressa* DNA and the 12 primer pairs. (B) Electrophoresis results following PCR using DNA from four constitutive green tide species in the Yellow Sea and the twelve primer pairs. Either “√” or “x” mean there are bands in the electrophoresis with related primers. Wherein “√” means the consistency of the band sequence with predicted tandem repeats, while “x” means not. “-” means no band; blank means not tested. Each primer pair was tested on two strains of the species in Panel A. Information about 12 pairs of primers was detailed in S3 Table in S1 File.

All the primer pairs designed across both sides of selected tandem repeats in the *U*. *compressa* extranuclear genome effectively performed in two strains of this species, as confirmed by gel electrophoresis (Fig 6A). After sequencing, products amplified with the primer pairs c-*psb*L-*psb*J, c-*rpo*C2, m-*cox*3-*trn*R, m-*trn*G-*trn*Y, and m-*trn*T-*trn*A-2 revealed the tandem repeat sequence predicted for *U*. *compressa*, while other primer pairs did not. Only one primer pair (c-*psb*L-*psb*J) successfully amplified the *U*. *flexuosa* genome (Fig 6B), but the resulting sequence was not as predicted.

**Fig 7:**
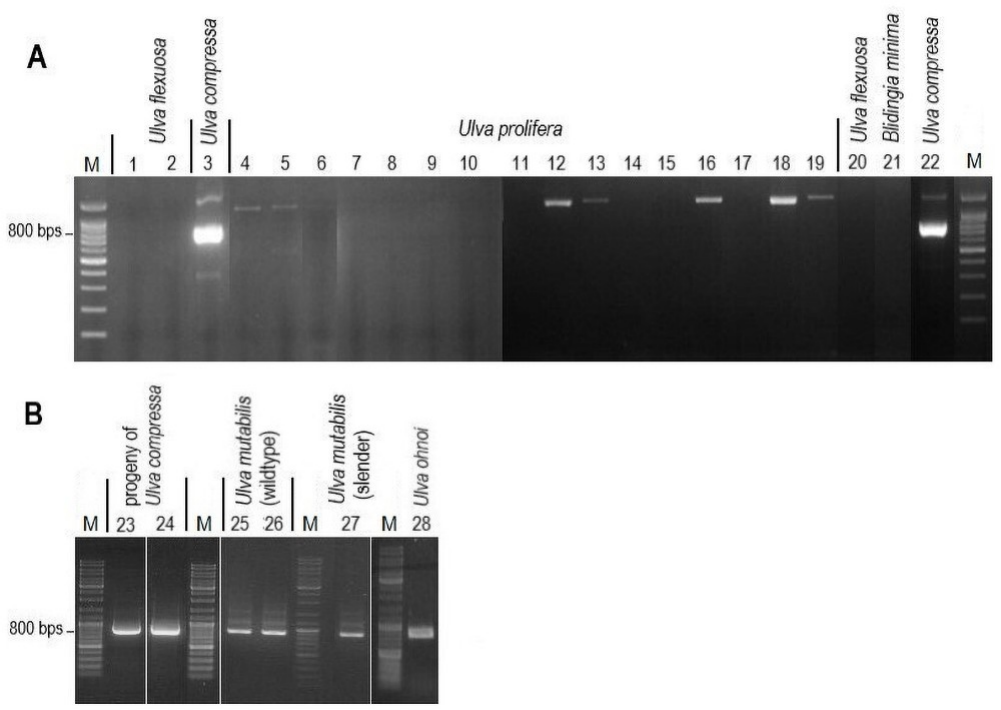
Application of the m-*trn*G-*trn*Y primer on *U*. *compressa* and *U*. *mutabilis*. (A) Lanes 1–22 correspond to amplification using m-*trn*G-*trn*Y with 22 green tide samples from the Yellow Sea, China, which were detailed in S4 Table in S1 File. Lanes 3 and 22 show the two brightest bands, characterized by the sequence GAAAATATAAATA (× 3), with a length of approximately 800 bp. In comparison with the DNA marker, the bands in lanes 4, 5, 12, 13,16, 18 and 19 were higher than 800 bp. Subsequent sequencing using the m-*trn*G-*trn*Y primer resulted in no result, suggesting the occurrence of non-specific amplification. (B) Lanes 23–28 correspond to PCR amplification using the m-*trn*G-*trn*Y primer with *U*. *compressa* progeny, the candidate model *U*. *mutabilis*, and *U*. *ohnoi*. Two different types of standard genetic markers were used in (A) and (B) because the two experiments were performed independently.

Following that, three primer pairs (m-*cox*3-*trn*R, m-*trn*G-trnY, and m-*trn*T-*trn*A-2) were tested on *U*. *linza* and *U*. *prolifera* to see if they generated PCR products from *U*. *compressa*, as evidenced by a distinct band on an agarose gel. The m-*trn*T-*trn*A-2 primer only worked for *U*. *prolifera*, did not reveal the predicted sequence (Fig 6B). When tested against all the strains collected from the Yellow Sea’s green tide (n = 22), the m-*trn*G-*trn*Y primer amplified the predicted ‘TRg’ sequence only in *U*. *compressa* and discriminated against *U*. *flexuosa*, *U*. *prolifera*, and *Blidingia minima* (S4 Table in S1 File and Fig 7A). As proof of principle, the TRg PCR product derived from *U*. *mutabilis* was conspecific to *U*. *compressa* and identical in size (800 bp), as predicted from the *U*. *mutabilis* genome [56] (Fig 7B).

Finally, the m-*cox*3-*trn*R primer produced non-specific bands in all samples when using green tide strains collected from the Yellow Sea (S4 Table in S1 File), which included *U*. *prolifera*, *U*. *compressa*, *U*. *flexuosa*, and *Blidingia minima*.

**Fig 8:**
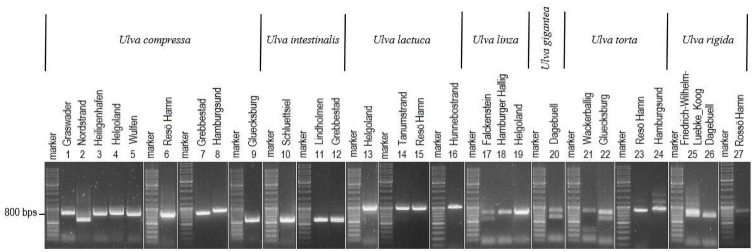
Application of primer m-*trn*G-*trn*Y on 27 *Ulva* samples collected in the North and Baltic Seas (Europe). The standard genetic marker is shown corresponding to the individual gel electrophoresis analysis. The name and number of the sampling site for each *Ulva* isolate are indicated above each lane. Details of the 27 samples are given in S5 Table in S1 File.

Samples from other regions reacted differently toward the specificity of the primers used. In addition to *U*. *compressa* and *U*. *mutabilis*, *U*. *ohnoi* (from the Mediterranean Sea) contained the same tandem repeat, as confirmed by sequencing analysis (S5 Fig in S1 File and Fig 7B). However, using the m-*trn*G-*trn*Y primer, amplification of samples collected in the North Sea and Baltic Sea (Europe) resulted in various PCR products of different lengths independent of the *Ulva* species (Fig 8). As a result, the species specificity of this primer could only be attributed to Yellow Sea strains.

## Discussion

### Comparative analysis of chloroplast genomes

The conspecificity of *U*. *compressa* and *U*. *mutabilis*, which has been studied separately for years [55, 59–61], was recently characterized using morphological patterns, hybridization, and molecular phylogenetic methods [17]. Thus, it was not surprising that the chloroplast genomes of *U*. *mutabilis* and *U*. *compressa* were consistent in their structure in terms of gene sequence and homology. Both species clustered together in the phylogenetic tree (Fig 4) and shared several forward/palindromic/tandem repeats, including TRg, detected in both the wild-type and slender-morphotype of *U*. *mutabilis*. However, *U*. *compressa* and *U mutabilis* (wild type) sequences differed in their total number of introns, length of intergenic regions (Fig 2), and transcription directions (S9 Table in S1 File), all of which were lesser conserved elements. The difference in the length of intergenic spacers may be caused by large-fragment insertions and deletions [62].

*Ulva prolifera*, *U*. *linza*, *U*. *compressa*, and *U*. *flexuosa* are the four main green-tide-forming *Ulva* species from the Yellow Sea that exhibit varying morphologies [35, 39]. *Ulva prolifera* and *U*. *linza* clustered together following whole chloroplast- and mitogenome analyses (compare with Fig 4). Analysis of the chloroplast genomes revealed that the *Ka* and *Ks* graphs generated from the selection force and substitution rate evaluation charts were almost identical (Fig 3). Furthermore, the sequence of complementary/forward/palindromic/tandem repeats was almost consistent (S10 and S11 Tables in S1 File). This observation could explain the phenomenon that *U*. *prolifera* was derived from the hybridization of *U*. *linza* and *U*. *procera* in the Yellow Sea [18].

*Ulva fasciata*, a heterotypic synonym of *U*. *lactuca* [17], shares a few genotyping markers with *U*. *ohnoi*, and is also closely related to *U*. *ohnoi* based on chloroplast genome analysis [63]. However, *tuf*A and *rbc*L analyses indicate that *U*. *fasciata* and *U*. *ohnoi* could be resolved into separate lineages. If more species were included in the analysis, they could be even separated by *U*. *spinulosa* and *U*. *reticulata* [64, 65]. *Ulva fasciata* and *U*. *ohnoi* shared only a few complementary/palindromic repeats and contained no forward or tandem repeats in their chloroplast genomes and mitogenomes. *U*. *mutabilis* and *U*. *compressa*, as well as and *U*. *prolifera* and *U*. *linza* contained several such repeats (S10 and S11 Tables in S1 File). Therefore, we suggest that the repeat sequences within intergenic regions in the extranuclear genome contain unique signals that characterize the species clustered together in the phylogenetic tree (Fig 4, S10 and S11 Tables in S1 File). The phylogenetic trees derived from chloroplast genomes and mitogenomes were highly similar.

The *Ka/Ks* ratios of most of the single coding genes indicated a purifying selection [14], which shows that the corresponding PCS continue to be consistent. Furthermore, the jagged curve along the gene queue in these circular DNA sequences was in line with the original transcription point, which was randomly distributed within the entire chloroplast genome, indicating a mutation in uniform distribution [66].

### Exploration of the *tuf*A and *pet*A markers

The mitogenomes and chloroplast genome contain potential barcodes [67–72]. *tuf*A is a genetic marker [16] with higher sensitivity and applicability than ITS, *rbc*L, universal amplicon, and large ribosomal subunit [18, 36, 37]. Moreover, *tuf*A can be used to characterize the evolutionary status of the extranuclear genome that has been confirmed in the present study by analyzing the aligned genes from chloroplast genomes and mitogenomes. *pet*A is one of the large subunits of the cytochrome b6-f complex that covalently binds one heme group and mediates electron transfer between photosystems II and I [73, 74]. In the present study, *pet*A possessed similar conserved molecular functions as *tuf*A owing to its key roles in cells and could, therefore, be used to verify the results obtained from *tuf*A-based phylogenetic analyses.

### Duplication of the non-coding sequence in extranuclear genomes

According to the endosymbiosis hypothesis, mitochondria originate from the aerobic bacteria [75], while chloroplasts come from endosymbiotic and photoautotrophic cyanobacteria [76–78]. However, the non-endosymbiotic hypothesis states that the mitochondria evolved from the membrane system such as the endoplasmic reticulum [79]. Both mitochondria and chloroplasts are organelles that contain DNA which is different from that of the nucleus but similar to that of the bacteria (i.e., covalent, closed, circular in shape, and similarly sized). The internal inheritance, differentiation, and gene flow transfer between the nuclear and extranuclear genomes in plants, animals, and bacteria are still being intensely studied [80, 81].

Compared to certain land plants, the genus *Ulva* has no large typical IR within the chloroplast genome to inhibit itself from undergoing gene recombination [82–84]; this could partly explain the introns presence in these prokaryote-derived chloroplasts [85]. Nevertheless, almost no recombination occurs between coding genes within the genus *Ulva*.

SSRs are valuable molecular markers with a high degree of intraspecific variation that are used in investigations focused on population genetics and polymorphism [86–89]. The present study results agreed with previous findings that reported that SSRs from chloroplast generally consist of short polyA or polyT repeats rather than tandem G or C repeats [90].

Based on the hypothesis of the endosymbiotic origin of chloroplasts [91], the non-species-specific IR (complementary/forward/palindromic) distributed among the chloroplast genomes of *Ulva* could be obtained in two ways: (1) We suggest that genes recombined in the ancestral cyanobacteria before entering relatively stable chloroplasts. (2) Alternatively, genes were acquired from the bacteria through horizontal gene transfer, as shown in the chloroplast genome of *Bryopsis plumosa* [92].

Specific spaces between coding genes are associated with the heterogeneity of evolutionary rates [93]. Examples include the *rnl-trn*K, *cox*3*-atp*6, and *trn*P*-rnl* spacers in the brown algae mitogenome [94], where tandem repeats enable the development of potential markers at various taxonomic levels [95]. The tandem repeats, which might be exclusive within the genus *Ulva*’s subspecies or neighboring species, are more abundant in the mitochondria than in the chloroplast. However, approximately half of all tested sequences in *U*. *compressa* did not contain the exact predicted tandem repeats owing to random or uneven variability, or fewer repeating numbers, which lowered its value as a potential species indicator (Fig 6). The fact that more intra-species shared tandem repeats instead of complementary/forward/palindromic repeats in fast-evolving mitogenomes might support the non-endogenous hypothesis that these tandem repeats are inherited from intracellular genetic material [79].

### Typical tandem repeats in the extranuclear genome

Although the tandem repeat sequence TRg is not unique to *U*. *compressa*, considering the whole analyzed data set, it could be used to accurately identify the organism from the major green tide forming *Ulva* species of the Yellow Sea using a non-sequencing approach.

Although no such TRg sequence exists in the chloroplast or mitochondrial genomes of *U*. *ohnoi*, TRg and related sequences of the nuclear genome were amplified, with which the corresponding sequence in *U*. *compressa*’s mitochondrial genome shared 99.8% homology. Considering endosymbiotic gene transfer [96, 97] in *Ulva* genomes, it is tempting to speculate that TRg originated from an ancestral mitogenome and integrated into the nuclear genome potentially via replicative transposition (*U*. *mutabilis*) or conservative transposition (*U*. *ohnoi*) as previously suggested.

Certain *U*. *compressa* strains sampled from the Baltic and North Sea contained primer-derived sequences which varied in lengths. This demonstrated that mutations (via insertion, deletion, or recombination) were common in the mitochondrial genome, or that they entered the nuclear genome via transposition, a phenomenon similar to that observed in the chloroplast genome [97–101]. Sequences of various length were amplified in *U*. *torta* and *U*. *rigida* using the same primers (Fig 8). The primer are thus not so conserved, and work for several *Ulva* species.

We determined that TRg was absent in the extranuclear genomes of *U*. *linza* and *U*. *fasciata* (*U*. *lactuca*). Thus, the amplicons might result from the respective nuclear genome sequences (Fig 8). Conservative transposition might explain this occurrence similar to *U*. *ohnoi* (Fig 7B). However, this phenomenon was not observed in *U*. *linza* collected from the Yellow Sea, where the TRg-related region may have been lost entirely or mutated in the primer region. The high reproduction rate that causes green tide blooms such in the Yellow Sea [102] might increase the probability of mutation events.

## Conclusions

In the present study, the complete chloroplast genome of *U*. *compressa* (RD9023) was sequenced and annotated, including its protein coding regions and repeat sequences. The study focused on assessing genome length, homology, gene order and direction, intron size, selection strength, and substitution rate. The generated phylogenetic tree was analyzed on the basis of single and aligned genes in the chloroplast genome compared to mitogenome genes of *Ulva* to detect evolutionary trends. We systematically screened for specific regions among representative fragments of all mitogenomes and chloroplast genomes of the *Ulva* gene for DNA barcoding purposes. Several strains from China and Europe have complemented the screening process. The chloroplast genomes of *U*. *mutabilis* and *U*. *compressa* shared the same gene queue and high homology of each gene and were clustered together in the phylogenetic tree. In addition, there were several forward/palindromic/tandem repeats in common, similar to those in *U*. *prolifera* and *U*. *linza*, but different from those in *U*. *fasciata* and *U*. *ohnoi*.

*Pet*A performed as an appropriate barcode marker gene following the results of the *tuf*A-based analysis. Furthermore, the chloroplast genome and mitogenomes have been compared. In agreement with the extranuclear genomes verified by the phylogenetic analysis of the aligned genes of both chloroplast genomes and mitogenomes, *pet*A showed the evolutionary trends of the reported species in the *Ulva* genome. Non-species-specific complementary/forward/palindromic interval repetitions have been more frequently distributed in chloroplast genomes than in mitogenomes. Conversely, tandem repetitions, which may be exclusive to neighboring species in the *Ulva* genus, were more abundant in mitochondria than in chloroplasts. The amplification of significant tandem repeats, such as TRg-related regions among mitogenomes, supported the identification of *U*. *mutabilis* and *U*. *compressa* within the green tide species sampled in this study of the Yellow Sea in China. However, this scenario was quite different for the European *Ulva* species, as this gene sequence varied across species boundaries and was not specific to the species collected in the North Sea and the Baltic Sea. It demonstrates the significance of *Ulva* sampling on a global scale for the development of a non-sequencing approach.

Our study is the foundation for future research on sequencing and characterization of other green tide algal species and screening for novel extranuclear markers.

Since there was a lack of strains in this study, more samples from different locations should be used to verify the results. It is also worth looking into the gene flow of high homology tandem repeats between nuclear and extranuclear genomes in future studies.

## Supporting information

S1 File (PDF)

S2 File (PDF)
