# Supplementary material for: Screening and verification of extranuclear genetic markers in green tide algae from the Yellow Sea
Source: PLoS One. 2021 Jun 1;16(6):e0250968. doi: 10.1371/journal.pone.0250968 (PMC8168861; doi:10.1371/journal.pone.0250968)

Supporting material for

Screening and verification of extranuclear genetic markers in green tide algae from the Yellow Sea

Chuner Cai[1,2*], Kai Gu[1], Hui Zhao[1], Sophie Steinhagen[3],

Peimin He[1], Thomas Wichard[2*]

[1]College of Marine Ecology and Environment, Shanghai Ocean University, Shanghai, China

[2]Institute for Inorganic and Analytical Chemistry, Jena School for Microbial Communication, Friedrich Schiller University Jena, Jena, Germany

[3]Department of Marine Sciences-Tjärnö Marine Laboratory, University of Gothenburg, Strömstad, Sweden

*** Corresponding authors**

E-mail

Chuner Cai: cecai@shou.edu.cn

Thomas Wichard: thomas.wichard@uni-jena.de

Content

Tables

Table S1. Primers used for sequencing the chloroplast genome from *U. compressa*

Table S2. Samples of green tides collected from the Yellow Sea, containing NCBI accession number of *tuf*Aand *pet*A as symbols

Table S3. Primers used in chloroplast genomes and mitogenome from *Ulva compressa* for the amplification of the tandem repeats

Table S4. Twenty-two green tide samples collected from the Yellow Sea during 2018-2019

Table S5. Twenty-seven samples in the genus *Ulva* collected from Europe

Table S6. Genes predicted in the chloroplast genome of *U. compressa*

Table S7. Distribution of tri-, tetra-, penta- and hexanucleotide SSR loci in the chloroplast genome of *U. compressa*

Table S8. Repeat sequences identified in the chloroplast genome of *U. compressa*.

Table S9. Transcription direction of seven PCGs and 14 *t*RNAs in the chloroplast genomes of *Ulva*

Table S10. Comparison of inverted repeat sequences such as complement/forward/palindrome repeats in *Ulva* chloroplast genomes and mitogenomes

Table S11. Comparison of tandem repeats in mitochondrial and chloroplast genomes among the genus *Ulva*.

Figures

Fig. S1. Phylogenetic analysis of strains used for chloroplast genome sequencing in this study and those from NCBI database according to *tuf*A and ITS

Fig. S2. Phylogenetic analysis using coding genes in chloroplast genomes in the genus *Ulva*

Fig. S3. Phylogenetic analysis of RNA sequences from the chloroplast genome of the genus*Ulva*

Fig. S4. Phylogenetic analysis of whole aligned *t*RNA sequence in the chloroplast genome of the genus *Ulva*.

Fig. S5. Phylogenetic analysis of *U. ohnoi* and those from the NCBI database according to *tuf*A.

Table S1. Primers used for sequencing the chloroplast genome from *Ulva compressa*.

label	primer	sequence	handling
BCP2	psbZ-1F/petA-1R	GTTTCATTATCTTTTATTCTTGTAATTGGTG/ATTGTCCACGACCTCTATTACCCC	breaking
BCP3	petA-1F/atpA-1R	CCTCAAGCAGTATTACCTGATAGTGTTTT/ GCTGAACCTACACGAGAAACAGAAATA	breaking
BCP9	psbB-1R/ycf3-1F	TGACTCGTTTAGGTGTTACCCAATCT/TTTACCAACATCAAATAGAGAAAAACAA	breaking
BCP12	psaA-1F/rpoC2-1R	GAAAAACATTGCTGCTGCCATTAC/ ATTGAACAACTTTTAGAAGCAAGAGAA	breaking
BCP14	rpoC2-2F/rpoB-1R	CTTTGTCCTGAAAAAACACCACCA/ GCTCGTTCAACTGGTCCTTATTCT	breaking
BCP7	petB-1F/rpl16-1R	CCTACTTGGTCCCAGGGTAATGA/TTCCCGATAAACCTGTAACAATGC	breaking
BCP6	psbE-1F/petB-1R	AAAAATGGCAGGGACTACAGGAGA/TTTATTATCGTCCAACAGTAGCAGAAG	breaking
BCP10	psbE-1R/psbA-1R	TGTAATACTGTGAATAACCCAATAACGA/TTTCAGTATTCATCATCGCATTTGTT	breaking
BCP13	rpoC2-1F/rpoC2-2R	CTTGAGATTGATAAACTTTTTGAACAGAA/AATCGTTTAGTTTCTTTAGGTGAATCTGT	breaking
BCP11	psbA-1F/psaA-1R	TGGGTAGAAGTGTAAACCAATAGCG/TAAATGGAGATGTAGGAGGCGGTT	breaking
BCP8	BCP-YCF3-1F/BCP-RPL16-1R	GCTCTACCATGTTTTCCGTTACTTG/ CGGCGTTGTTACTCCTCTAATTTC	breaking
BCP4	BCP-PSBD-1R/BCP-ATPA-1F	CCCATAGAGTTTGGCGGTGTT/ GAAACTCAAGAAGGTGATGTATCAGC	breaking
BCP17	BCP-RPS4-1F/BCP-FTSH-1R	CACCCGTTGAACCGTTTGAT/ TGCTCCACCTGCTTTTGAATACT	breaking
BCP15	BCP-RPS4-1R/BCP-RPOC1-1F	GGACCTCGTTTAAGATTAGTTCGTAA/TTTCTTCCTTCAATATCATCCGATT	breaking
BCP67	BCP-psbC-1F/BCP-PSBB-1R	GCAATGAATCCCATTAAAGAAATAG/CACATAACCCAATGCCAGATAGC	sequencing

Table S2. Samples of green tides collected from the Yellow Sea with NCBI accession number for *tufa* and *pet*A. (NCBI/GenBank accession numbers are given).

Biomaterial	species	*tuf*A	*pet*A	date	Location
*Ulva*_2428	*U. compressa*	MN560983	MN524517	2016	floating in Rudong sea area
*Ulva*_2427	*U. compressa*	MN560984	MN524518	2016	floating in Rudong sea area
*Ulva*_2424	*U. prolifera*	MN560985	MN524519	2016	floating in Rudong sea area
*Ulva*_2423	*U. prolifera*	MN560986	MN524520	2016	floating in Rudong sea area
*Ulva*_2425	*U. linza*	MN560987	MN524521	2016	floating in Rudong sea area
*Ulva*_2426	*U. linza*	MN560988	MN524522	2016	floating in Rudong sea area
*Ulva*_2429	*U. flexuosa*	MN560989	MN524523	2016	floating in Rudong sea area
*Ulva*_2430	*U. flexuosa*	MN560990	MN524524	2016	floating in Rudong sea area
*Ulva*_3669	*U. prolifera*	MN560991	MN524525	25-Apr-2018	truss in Rudong sea area
*Ulva*_3670	*U. prolifera*	MN560992	MN524526	9-Jul-2019	floating in Qingdao sea area
*Ulva*_3674	*U. prolifera*	MN560993	MN524527	24-Apr-2018	truss in Qingdao
*Ulva*_3675	*U. flexuosa*	MN560994	MN524528	24-Apr-2018	floating on the shore of Qidong
*Ulva*_3677	*U. compressa*	MN560995	MN524529	13-Apr-2019	truss at Jiangjiasha sea area
*Ulva*_3678	*U. prolifera*	MN560996	MN524530	2-Jun-2019	Yellow Sea 33.11 N 121.11E
*Ulva*_3679	*U. prolifera*	MN560997	MN524531	3-Jun-2019	Yellow Sea 33.11 N 121.11E
*Ulva*_3680	*U. prolifera*	MN560998	MN524532	12-Jun-2019	Yellow Sea 32.42 N 121.24E
*Ulva*_3681	*U. prolifera*	MN560999	MN524533	12-Jun-2019	Yellow Sea 32.42 N 121.24E
*Ulva*_3682	*U. compressa*	MN561000	MN524534	4-Jun-2019	truss in Rudong sea area
*Ulva*_3683	*U. prolifera*	MN561001	MN524535	2-Jun-2019	floating in the south Yellow Sea
*Ulva*_3684	*U. prolifera*	MN561002	MN524536	Jun-2019	floating in the south Yellow Sea
*Ulva*_3685	*U. prolifera*	MN561003	MN524537	Jun-2019	floating in the south Yellow Sea
*Ulva*_3686	*U. prolifera*	MN561004	MN524538	15-Jul-2019	floating in the south Yellow Sea
*Ulva*_3687	*U. flexuosa*	MN561005	MN524539	2-Jun-2018	YJ42（south Yellow Sea）

**Biomaterial**	**species**	***tuf*A**	***pet*A**	**date**	**Location**
*Ulva*_3689	*U. prolifera*	MN561006	MN524540	May-2018	floating in the south Yellow Sea
*Ulva*_3690	*U. prolifera*	MN561007	MN524541	Mar-2019	floating in the south Yellow Sea
*Ulva*_3691	*U. prolifera*	MN561008	MN524542	Mar-2019	floating in the south Yellow Sea
*Ulva*_3692	*U. prolifera*	MN561009	MN524543	Mar-2019	floating in the south Yellow Sea

Table S3. Primers used in chloroplast genomes and mitogenome from *Ulva compressa* for the amplification of the tandem repeats.

Genome	Repeat unit sequence	Primer	Primers sequence (Forward primer/ Reverse primer)	Molecular weight (bps)
chloroplast	AATATA(x4)	c-ycf20-ccsA	AGGTCCAGAAACAACTCGTAG/GGAAATGGTTTGAATTTACCCAACG	400-450
	TTTAATTATTA(x3)	c-psbL-psbJ	ACCAAACCCAAATAAACAAACTGT/ GCAGCAACTCCTACTACAACAC	350
	AATTTAAAACAA(x2)	c-rpoC2	GCAGGGTAGTTTGCTTTTGGG/ TTGAGCAAGGAACAAAATCACC	500-550
Mitochondria	ACTAATAAAT(x5)	m-trnT-trnA-1	GCAACTCGGGAATTGAACCT/ GAAACCCAAACGCTACGCTAC	600-650
	GGCAAAAGCAAA(x2)	m-cox3-trnR	GCAGTACATCTCGCCACCAT/ GGCAAAGGCAAAAGGCACAT	450
	TTAAACTATTAA(x2)	m-cox2-nad5	GAACCTTTGCTTTCGCCCTT/ GGGGCAACTCAATCTGGCTT	400-450
	GAAAATATAAATA(x3)	m-trnG-trnY	TGCGGCTATGGGCTATTGATT/ AGTCCTAAGCCTGCAATGGT	800
	CCCTTGCCTTTGGGC(x2)	m-rps14	CCGGCGTAAGGTAAGCTGTT/ TTACGTGACCTTTGCCCCC	550
	AAAATCTAAAATCTAAAA(x2)	m-trnT-trnA-2	AGGCCTAAGACTTTACCGCC/ AAGGACAGTGAAGACCTGCG	600-650
	CCCGCACAGCACCTTTGGC(x2)	m-trnT-rrl	CTCTAACCTTTGCCCTTGCCT/ GTAAATAGGCGGAGGGGTGT	300
	GGCCAAAGGTGCTGTGCGG(x2)	m-trnI-cob	GGCCCGAACCTTTGCTTTTG/ AGGGCAAACGGCTACACTAT	200-300
	TTAGTATGTTGTTTGTTTAACTGA(x2)	m-rpl14-cox3	TTTTACGCCCGCTGCTTTTT/ GCAGCAACTTTTGCCTTTGC	900

Table S4. Twenty-two green tide samples collected from the Yellow Sea during 2018-2019.

No.	Biomaterial	Species	date	Location
1	*Ulva*_3672	*U. flexuosa*	25-Apr-2018	truss at Rudong sea area
2	*Ulva*_3675	*U. flexuosa*	24-Apr-2018	floating on the shore of Qidong
3	*Ulva*_3677	*U. compressa*	13-Apr-2019	truss at Jiangjiasha sea area
4	*Ulva*_3669	*U. prolifera*	25-Apr-2018	truss in Rudong sea area
5	*Ulva*_3670	*U. prolifera*	9-Jul-2019	floating in Qingdao sea area
6	*Ulva*_3674	*U. prolifera*	24-Apr-2018	truss in Qingdao
7	*Ulva*_3678	*U. prolifera*	2-Jun-2019	Yellow Sea 33.11 N 121.11E
8	*Ulva*_3679	*U. prolifera*	3-Jun-2019	Yellow Sea 33.11 N 121.11E
9	*Ulva*_3680	*U. prolifera*	12-Jun-2019	Yellow Sea 32.42 N 121.24E
10	*Ulva*_3681	*U. prolifera*	12-Jun-2019	Yellow Sea 32.42 N 121.24E
11	*Ulva*_3683	*U. prolifera*	2-Jun-2019	Floating in the South Yellow Sea
12	*Ulva*_3684	*U. prolifera*	Jun-2019	Floating in the South Yellow Sea
13	*Ulva*_3685	*U. prolifera*	Jun-2019	Floating in the South Yellow Sea
14	*Ulva*_3686	*U. prolifera*	15-Jul-2019	Floating in Rizhao sea area
15	*Ulva*_3689	*U. prolifera*	May-2018	Floating in the South Yellow Sea
16	*Ulva*_3690	*U. prolifera*	Mar-2019	Floating in the South Yellow Sea
17	*Ulva*_3691	*U. prolifera*	Mar-2019	Floating in the South Yellow Sea
18	*Ulva*_3692	*U. prolifera*	Mar-2019	Floating in the South Yellow Sea
19	*Ulva*_3693	*U. prolifera*	Mar-2019	Floating in the South Yellow Sea
20	*Ulva*_3687	*U. flexuosa*	2-Jun-2018	YJ42（South Yellow Sea）
21	*Ulva*_3694	*Blidingia minima*	Mar-2019	Floating in the South Yellow Sea
22	*Ulva*_3682	*U. compressa*	4-Jun-2019	truss in Rudong sea area

Table S5. **Twenty-seven samples of the genus *Ulva* collected in Europe.**

Lane	Sample No.	Date	Location	Latitude and longitude	species
1	S_362	2014/8/26	Schleswig-Holstein, Heiligenhafen natural reserve ´Graswader´	N 54°22.680; E 011°00.515	*U. compressa*
2	S_651	2015/4/21	Schleswig-Holstein, Nordstrand	N54°28.243; E 008°.48.410	*U. compressa*
3	S_626	2015/4/16	Schleswig-Holstein, Heiligenhafen inland lake	N 54°22.723; E 010°57.327	*U. compressa*
4	S_6	2014/7/22	Helgoland	N 54°10.189; E 007°53.365	*U. compressa*
5	S_514_B	2014/9/17	Wulfen	N 54°24.535; E 011°10.388	*U. compressa*
6	SV 16.1	Sep-18	Resö Hamn	N 58.7999; E 11.1654	*U. compressa*
7	SV 23	Sep-18	Grebbestad	N 58.6836; E 11.258	*U. compressa*
8	SV 33.1	Sep-18	Hamburgsund	N 58.5527; E 11.2683	*U. compressa*
9	S_72	2014/7/24	Glücksburg	N 54°50.205; E 009°31.382	*U. intestinalis*
10	S_133	2014/7/31	Schlüttsiel	N 54°40.904; E 008°45.249	*U. intestinalis*
11	SV 46.1	2019/1/20	Lindholmen	N 58.8914; E 11.13342	*U. intestinalis*
12	SV 75	2019/2/13	Grebbestad	N 58.6836; E 11.258	*U. intestinalis*
13	S_729	2015/4/24	Helgoland	N 54°10.937; E 007°53.301	*U. lactuca*
14	SV 26.1	Sep-18	Tanumstrand	N 58.6721; E 11.2622	*U. lactuca*
15	Sv 15.1	Sep-18	Resö Hamn	N 58.7999; E 11.1654	*U. lactuca*
16	SV 44	Sep-18	Hunnebostrand	N 58.4365; E 11.29	*U. lactuca*
17	S_241_*U.linza* 1	2014/8/18	Falckenstein	N 54°39.368; E 010°18.960	*U. linza 1*
18	S_504_*U.linza*_1	2014/9/16	Hamburger Hallig	N 54°35.939; E 008°48.730	*U. linza 1*
19	S_727_*U.linza*_2	2015/4/24	Helgoland	N 54°10.937; E 007°53.301	*U. linza 2*
20	S_632	2015/4/17	Dagebuell	N 54°43.804; E 008°41.35	*U. gigantea*
21	S_81	2014/7/24	Wackerballig	N 54°45.518; E 009°52.670	*U. torta*
22	S_73	2014/7/24	Glücksburg	N 54°50.205; E 009°31.382	*U. torta*
23	SV 14	Sep-18	Resö Hamn	N 58.7999 ; E 11.1654	*U. torta*

Lane	Sample No.	Date	Location	Latitude and longitude	species
24	SV 32	Sep-18	Hamburgsund	N 58.5527 ; E 11.2683	*U. torta*
25	S_449	2014/9/9	Friedrich-Wilhelm-Lübke_Koog	N 54°49.998 ; E 008°36.852	*U. rigida*
26	S_123	2014/7/30	Dagebüll	N 54°43.804 ; E 008°41.35	*U. rigida*
27	SV 11.1	Sep-18	Rossö Hamn	N 58.8563 ; E 11.1713	*U. rigida*

Table S6. Genes predicted in the chloroplast genome of *U. compressa*

Category of genes	Group of genes	Name of genes
**Self-replication**	tRNA genes	26 *trn* genes
	Small subunit of the ribosome	*rps*11, *rps*12, *rps*14, *rps*18, *rps*19, *rps*2, *rps*3, *rps*4, *rps*7, *rps*8, and *rps*9
	Large subunit of the ribosome	*rpl*12, *rpl*14, *rpl*16, *rpl*19, *rpl*2, *rpl*20, *rpl*23, *rpl*32, *rpl*36, and *rpl*5
	DNA dependent RNA polymerase	*rpo*A, *rpo*B, *rpo*C1, and *rpo*C2
	rRNA genes	*rrl*(23S), *rrn* (5S) and *rrs*(16S)
**Genes for photosynthesis**	Subunits of ATP synthase	*atp*A, *atp*B, *atp*E, *atp*F, *atp*H, and*atp*I
	Subunits of cytochrome b/f complex	*pet*A, *pet*B, *pet*D, *pet*G, and*pet*L
	Subunits of photosystem I	*psa*A, *psa*B, *psa*C, *psa*I, *psa*J, *psa*M, *ycf*3, and *ycf*4
	Subunits of photosystem II	*psb*A, *psb*B, *psb*C, *psb*D, *psb*E, and*psb*F, *psb*H, *psb*I, *psb*J, *psb*K, *psb*L, *psb*M, *psb*N, *psb*T, *psb*Z, and *ycf*12
	Subunit of rubisco	*rbc*L
	Subunit of protochlorophyllide reductase	*chl*I
**Other genes**	Subunits of Acetyl-CoA-carboxylase	*acc*D
	C-type cytochrome synthesis gene	*ccs*A
	Envelope membrane protein	*cem*A
	Protease	*clp*P
	Elongation factor	*tuf*A
	Zinc metalloprotease	*fts*H
	Translation initiation factor	*inf*A
**Genes of unknown function**	Conserved open reading frames	*ycf*1,and *ycf*20

Table S7. Distribution of tri-, tetra-, penta- and hexanucleotide SSR loci in the chloroplast genome of *U. compressa*. a: Intergenic spacer region, b: Coding sequences.

SSR type	SSR sequence	Start	End	Location
tri	$(TAT)_4$	5674	5685	IGS[a](*trnF-petD*)
tri	$(ATT)_4$	29887	29898	CDS[b](*psbD*)
tri	$(ATT)_4$	32302	32313	IGS(*trnM-trnD*)
tri	$(ATA)_4$	32338	32349	IGS(*trnM-trnD*)
tri	$(ATA)_4$	39907	39918	IGS(*atpH-atpI*)
tri	$(AAT)_4$	51711	51722	IGS(*trnC-trnE*)
tri	$(AAT)_4$	54408	54419	IGS(*rpl32-chlI*)
tri	$(AAT)_4$	64968	64979	CDS(*rpoC1*)
tri	$(TAA)_4$	65324	65335	CDS(*rpoC1*)
tri	$(TAA)_4$	65639	65650	CDS(*rpoC1*)
tri	$(TAA)_4$	66627	66638	CDS(*rpoC1*)
tri	$(TAA)_4$	71620	71631	CDS(*rpoC2*)
tri	$(ATA)_4$	72111	72122	CDS(*rpoC2*)
tri	$(AAT)_4$	73100	73111	CDS(*rpoC2*)
tri	$(TAA)_4$	74299	74310	CDS(*rpoC2*)
tri	$(TAA)_4$	76447	76458	CDS(*rpoC2*)
tri	$(ATA)_4$	85401	85412	IGS(*rrl-psaA*)
tri	$(CAA)_4$	85678	85689	CDS(*psaA*)
tri	$(TAA)_4$	94499	94510	IGS(*psbA-trnT*)
tetra	$(AATT)_3$	14286	14297	IGS(*infA-rps8*)
tetra	$(ATTA)_3$	26345	26356	CDS(*psbN*),IGS(*psbN-psbH*)
tetra	$(CTTT)_3$	28707	28718	IGS(*psbB-psbD*)
tetra	$(TTAA)_3$	32686	32697	IGS(*trnD-psaB*)
tetra	$(TAAT)_3$	35307	35318	IGS(*trnH-trnS*)
tetra	$(TTAT)_3$	50120	50131	IGS(*trnK-trnY*)
tetra	$(ATTT)_3$	51511	51522	IGS(*trnC-trnE*)
tetra	$(TATT)_3$	54289	54300	IGS(*rpl32-chlI*)
tetra	$(TTTA)_3$	55764	55775	IGS(*psaJ-trnW*)
tetra	$(AATT)_3$	58483	58494	CDS(*rpl12*),IGS(*rpl12-trnV*)
tetra	$(GTAG)_3$	81998	82009	CDS(*rrl*)
tetra	$(ATAA)_3$	87880	87891	IGS(*psaA-psaC*)
tetra	$(TTTA)_3$	88010	88021	IGS(*psaA-psaC*)
penta	$(ATTAT)_3$	28768	28782	IGS(*psbB-psbD*)
penta	$(CTCGT)_3$	41744	41758	IGS(*rps2-trnS*)
penta	$(TCGTC)_3$	41760	41774	IGS(*rps2-trnS*)
hexa	$(GGGTCA)_3$	57880	57897	IGS(*rps9-rpl12*)
hexa	$(TAAAAA)_3$	62062	62079	CDS(*rpoB*)
hexa	$(TGGATA)_3$	65809	65826	CDS(*rpoC1*)
hexa	$(ATATGG)_3$	71820	71837	CDS(*rpoC2*)

SSR type	SSR sequence	Start	End	Location
hexa	$(GGTCAT)_4$	71870	71893	CDS(*rpoC2*)
hexa	$(TGGATA)_3$	74979	74996	CDS(*rpoC2*)
hexa	$(TTTGGT)_3$	85359	85376	IGS(*rrl-psaA*)
hexa	$(AATATA)_4$	88925	88948	IGS(*ycf20-ccsA*)

Table S8. Repeat sequences identified in the chloroplast genome of *U. compressa*. Types T and F refer to the tandem repeats and forward repeats, respectively. a: Intergenic spacers, and b: Coding sequences.

Repeat size (bp)	Type	Location	Repeat unit sequence
33	T	IGS[a](*psbL-psbJ*)	(TTTAATTATTA) $_3$
32	T	IGS(*infA-rps8*)	(TTTATACTCCCCTGCT)$_2$
36	T	IGS(*infA-rps8*)	(TAATGTTTTTAAATTAAT)$_2$
66	T	IGS(*trnP-cemA*)	(TATATAATTTTTTACACTATTATCAAATACTAA)$_2$
30	T	IGS(*trnL-rps12*)	(CAGAGGGTATAAAAG)$_2$
30	T	IGS(*rps2-trnS*)	(CTCGT)$_6$
36	T	CDS[b](*ftsH*)	(AATTAAAAATTA)$_3$
24	T	IGS(*accD-clpP*)	(ATTTTTTTTATTA)$_2$
24	T	IGS(*ftsH-psbZ*)	(TTTATTATATTAA)$_2$
50	T	IGS(*trnE-trnM*)	(AATACTAATATAATATTAACTTTTT)$_2$
30	T	IGS(*rps9-rpl12*)	(CTGC-TTTATACCCT)$_2$
34	T	CDS(*rpoC1*)	(TAAAAAAATTTATAAAT)$_2$
24	T	CDS(*rpoC1*)	(CCCAAGCCCAAG)$_2$
32	T	CDS(*rpoC2*)	(ATTTTATTTTTACTAA)$_2$
24	T	CDS(*rpoC2*)	(AATTTAAAACAA)$_2$
28	T	IGS(*petD-petB*)	(TAATATATATTAAT)$_2$
32	T	IGS(*psaA-psaC*)	(TGTTTTATTAATTATT)$_2$
24	T	IGS(*ycf20-ccsA*)	(AATATA)$_4$
63	F	CDS(*psaB*), CDS(*psaA*)	GCACACCATCATTTAGCAATTGCTGTCTTATTTATTGTAGCAGGTCATATGTACCGTACTAAT
49	F	IGS(*infA-rps8*), IGS(*rrl-psaA*)	CTGCTTTTATACCCTCTGCGAATACTCAGAGGATTAACCTTAAGCAAAA
47	F	IGS(*infA-rps8*), IGS(*rps9-rpl12*)	TTTATACCCTCTGCTTTTATACCCTCTGCGAATACTCAGAGGATTAA

Repeat size (bp)	Type	Location	Repeat unit sequence
39	F	IGS(*infA-rps8*), IGS(*rrl-psaA*)	TTGCTTTATATTTATACTCCCCTGCTTTTATACCCTCTG
37	F	IGS(*rps9-rpl12*), IGS(*rrl-psaA*)	CTGCTTTTATACCCTCTGCGAATACTCAGAGGATTAA
37	F	CDS(*rpoC1*), IGS(*tufA-rps14*)	TGGATATTTCCGAACCTTTGGATATGGATATTCCCGA
35	F	IGS(*rpl32-chlI*), IGS(*rpl32-chlI*)	AAAATTATATTATAGATAATTTATTATCTATAAAT
33	F	CDS(*rpoC1*), CDS(*rpoC2*)	CTTTCCTTTGGATATGGATATGGATATTTCCGA
31	F	CDS(*rpoB*), IGS(*rrl-psaA*)	GCTTTTCTTTTGCCCTTGCAAAAGCAAGGGG
31	F	CDS(*ccsA*), CDS(*ycf1*)	CCTTTTCTTTTGATTACTCAAAGGATTCATC

Table S9. Transcription direction of seven PCGs and 14 *t*RNAs in the chloroplast genomes of *Ulva*. FD for forward direction and RC means reverse complement (gene is on the complementary strand).

	U. compressa	***U. mutabilis***	***U. flexuosa***	***U. linza***	***U. prolifera***	***U. ohnoi***	***U. fasciata***
*trn*G-UCC	FD	FD	FD	FD	FD	RC	RC
*trn*P-GGG	FD	FD	RC	FD	FD	RC	RC
*cem*A	FD	RC	RC	RC	RC	RC	RC
*ycf*4	FD	RC	RC	RC	RC	RC	RC
*clp*P	FD	RC	RC	RC	RC	RC	RC
*trn*F	FD	FD	FD	FD	FD	RC	RC
*rpl*36	RC	FD	RC	RC	RC	RC	RC
*trn*L_UAA	RC	FD	RC	FD	FD	FD	RC
*trn*M_1	FD	FD	FD	FD	FD	RC	RC
*psb*D	FD	FD	RC	RC	RC	RC	RC
*psb*C	FD	FD	RC	RC	RC	RC	RC
*trn*L_UAG	FD	FD	FD	RC	FD	FD	FD
*trn*N_GUU	RC	FD	FD	FD	FD	FD	FD
*pet*G	FD	RC	RC	RC	RC	RC	RC
*trn*K_UUU	RC	FD	RC	FD	FD	RC	RC
*trn*M_3	RC	FD	RC	FD	FD	RC	RC
*trn*Q_UUG	RC	FD	RC	FD	FD	RC	FD
*trn*W_CCA	FD	FD	FD	FD	FD	RC	RC
*trn*V_UAC	RC	FD	RC	FD	FD	RC	RC
*trn*R	FD	FD	FD	FD	FD	RC	RC
*trn*T_UGU	RC	FD	RC	FD	FD	RC	RC

Table S10. Comparison of inverted repeat sequences (IR) such as complement/forward/palindrome repeats in *Ulva* chloroplast genomes (over 20 bps) and mitogenomes (over 30 bps). a. *U. compressa,* **b**. *U. mutabilis,* **c**. *U. flexuosa,* **d**. *U. linza,* **e**. *U. prolifera,* **f**. *U. ohnoi*, and **g**. *U. fasciata.*

**Type**		**No.**	**Sequence (base number)**	**a**	**b**	**c**	**d**	**e**	**f**	**g**
chloroplast genomes	complement match	1	ATTATATAAATTAAAATAATTTTTT (24)			√			√	√
		2	ATTATATAAATTAAAATAATTTTTTG (25)				√	√		
	forward match	3	TAACAATTAATTAAATATACTTTATATAAA (29)				√	√		
		4	AGTTTCCCTTGTGATGGTCCAGGTCGTGGTGGAACATGT (38)				√	√	√	
		5	GGCACACCATCATTTAGCAATTGCTGTCTTATTTATTGTAGCAGGTCATATGTACCGTAC TAATT (64)	√	√					
	palindromic match	6	TATATTATTTTATATTATTTTATCTT (25)			√	√			
		7	CAATATAAACTATACTAAAACACTTAA (26)						√	√
		8	ATTAAAAAATATTTTATTTTAATTTTTA (27)				√	√		
		9	TTTAATATAGATTGTAATAAAACACTTAA (28)				√	√		
		10	TTAAAATTATAATGATTGTCAATTTTTATATT (31)			√	√	√		
		11	TTTTAATTAATATGTATTTATATATTTAATTAA (32)	√	√					
		12	ATTAATTAATATTTTATTTTAATTTTTATACAAA (33)	√	√					
		13	CAGAACCGTACGTGAGACTTTCACCTCATACGGCTCC (36)	√					√	
mitogenome genomes forward match		14	AAAAAGTGGTCTTTTTTGTTATAAAACAGCTTGTATAGAGAAGCGATGCTTTTGCCTTT GCCTGCCAAAAGAAACATAAAGCAG (84)				√	√		

Table S11 Comparison of tandem repeats in mitochondrial and chloroplast genomes among the genus *Ulva*.

Note: * means the same repeats at another site. (NCBI/GenBank accession numbers are given.)

In mitochondrial genome	Accession	Species	Repeat size (bp)	Repeat unit sequence	Copy number
	KX595276	*U. compressa*	10	ACTAATAAAT	5
	WT_177	*U. mutabilis*	10	ACTAATAAAT	5
	KX595276	*U. compressa*	12	GGCAAAAGCAAA	2
	WT_177	*U. mutabilis*	12	GGCAAAAGCAAA	2
	KX595276	*U. compressa*	12	TTAAACTATTAA	2
	WT_177	*U. mutabilis*	12	TTAAACTATTAA	2
	**KX595276**	***U. compressa***	**13**	**GAAAATATAAATA (abbreviated as TRg)**	**3**
	WT_177	*U. mutabilis*	13	GAAAATATAAATA	3
	KX595276	*U. compressa*	15	CCCTTGCCTTTGGGC	2
9 groups	WT_177	*U. mutabilis*	15	CCCTTGCCTTTGGGC	2
	KX595276	*U. compressa*	18	AAAATCTAAAATCTAAAA	2
	WT_177	*U. mutabilis*	18	AAAATCTAAAATCTAAAA	2
	KX595276	*U. compressa*	19	CCCGCACAGCACCTTTGGC	2
	NC_041082	*U. compressa*	19	CCCGCACAGCACCTTTGGC	2
	WT_177	*U. mutabilis*	19	CCCGCACAGCACCTTTGGC	2
	KX595276	*U. compressa*	19	GGCCAAAGGTGCTGTGCGG	2
	NC_041082	*U. compressa*	19	GGCCAAAGGTGCTGTGCGG	2
	WT_177	*U. mutabilis*	19	GGCCAAAGGTGCTGTGCGG	2
	KX595276	*U. compressa*	24	TTAGTATGTTGTTTGTTTAACTGA	2

In mitochondrial genome	Accession	Species	Repeat size (bp)	Repeat unit sequence	Copy number
	WT_177	*U. mutabilis*	24	TTAGTATGTTGTTTGTTTAACTGA	2
7 groups	KU182748	*U. fasciata*	8	ATCATAAA	3
	NC_028081	*U. fasciata*	8	ATCATAAA	3
	KU182748	*U. fasciata*	9	TATATAAGA	3
	NC_028081	*U. fasciata*	9	TATATAAGA	3
	KU182748	*U. fasciata*	12	GAAAATAAATTC	3
	NC_028081	*U. fasciata*	12	GAAAATAAATTC	3
	KU182748	*U. fasciata*	13	ATAAAATAATTTG	3
	NC_028081	*U. fasciata*	13	ATAAAATAATTTG	3
	KU182748	*U. fasciata*	13	TCTTAATTGGCTT	2
	NC_028081	*U. fasciata*	13	TCTTAATTGGCTT	2
	KU182748	*U. fasciata*	13	TTGTGTTTAAAAC	2
	NC_028081	*U. fasciata*	13	TTGTGTTTAAAAC	2
	KU182748	*U. fasciata*	**35**	ATTATTTTTATCTTTGAATGAAGTCTCAATTAATA	2
	NC_028081	*U. fasciata*	**35**	ATTATTTTTATCTTTGAATGAAGTCTCAATTAATA	2
7 groups	KY626326	*U. flexuosa*	11	AACCTTTGCTT	3
	NC_035809	*U. flexuosa*	11	AACCTTTGCTT	2
	KY626326	*U. flexuosa*	11	GCAAAGGTTAA	3
	KY626326	*U. flexuosa*	11	GCAAAGGTTAA	5
	KY626326	*U. flexuosa*	11	GCAAAGGTTAA	3*
	NC_035809	*U. flexuosa*	11	GCAAAGGTTAA	2

In mitochondrial genome	Accession	Species	Repeat size (bp)	Repeat unit sequence	Copy number
	NC_035809	*U. flexuosa*	11	GCAAAGGTTAA	2*
	NC_035809	*U. flexuosa*	11	GCAAAGGTTAA	4
	KY626326	*U. flexuosa*	11	TAACCTTTGCT	7
	NC_035809	*U. flexuosa*	11	TAACCTTTGCT	6
	KY626326	*U. flexuosa*	15	ATAATCTTAGCCTAC	2
	NC_035809	*U. flexuosa*	15	ATAATCTTAGCCTAC	2
	KY626326	*U. flexuosa*	15	CCCCTTGCCTTTGGC	2
	NC_035809	*U. flexuosa*	15	CCCCTTGCCTTTGGC	2
	KY626326	*U. flexuosa*	18	TTCAAAACTACTAAACAA	2
	NC_035809	*U. flexuosa*	18	TTCAAAACTACTAAACAA	2
	KY626326	*U. flexuosa*	19	TTATTTAGGCCTATGAAAG	2
	NC_035809	*U. flexuosa*	19	TTATTTAGGCCTATGAAAG	2
	KX530817	*U. pertusa*	12	ATTATTTTAACT	4
	NC_035722	*U. pertusa*	12	ATTATTTTAACT	4
	KX530817	*U. pertusa*	14	TAATTAATAATCAA	2
	NC_035722	*U. pertusa*	14	TAATTAATAATCAA	2
8 groups	KX530817	*U. pertusa*	16	TTTTATAAGAAGGTGA	3
	NC_035722	*U. pertusa*	16	TTTTATAAGAAGGTGA	3
	KX530817	*U. pertusa*	17	TACAAAATACCTTAATG	16
	NC_035722	*U. pertusa*	17	TACAAAATACCTTAATG	11
	KX530817	*U. pertusa*	20	ATTTTAATTTACATTAAAGG	2

In mitochondrial genome	Accession	Species	Repeat size (bp)	Repeat unit sequence	Copy number
	NC_035722	*U. pertusa*	20	ATTTTAATTTACATTAAAGG	2
	KX530817	*U. pertusa*	20	TTACTACGATTATATAAAAT	2
	NC_035722	*U. pertusa*	20	TTACTACGATTATATAAAAT	2
	KX530817	*U. pertusa*	21	ATATAAAAGCATAAATAATAA	2
	NC_035722	*U. pertusa*	21	ATATAAAAGCATAAATAATAA	2
	KX530817	*U. pertusa*	26	TTTTATCATGCAAAGCAAACACAACA	2
	NC_035722	*U. pertusa*	26	TTTTATCATGCAAAGCAAACACAACA	2
8 groups	NC_029701	*U. linza*	11	TATCTTATTAA	14
	KU161104	*U. prolifera*	11	TATCTTATTAA	**20**
	NC_028538	*U. prolifera*	11	TATCTTATTAA	**20**
	NC_029701	*U. linza*	12	ATAGATATCGAA	2
	KU161104	*U. prolifera*	12	ATAGATATCGAA	2
	NC_028538	*U. prolifera*	12	ATAGATATCGAA	2
	NC_029701	*U. linza*	14	AAGCCCTTATAATG	2
	KU161104	*U. prolifera*	14	AAGCCCTTATAATG	2
	NC_028538	*U. prolifera*	14	AAGCCCTTATAATG	2
	NC_029701	*U. linza*	18	TTTGGCTTCTTTGAAAGG	2
	KU161104	*U. prolifera*	18	TTTGGCTTCTTTGAAAGG	2
	NC_028538	*U. prolifera*	18	TTTGGCTTCTTTGAAAGG	2
	NC_029701	*U. linza*	20	ATTATAAAGACCTAAATAAA	2
	KU161104	*U. prolifera*	20	ATTATAAAGACCTAAATAAA	2

In mitochondrial genome	Accession	Species	Repeat size (bp)	Repeat unit sequence	Copy number
	NC_028538	*U. prolifera*	20	ATTATAAAGACCTAAATAAA	2
	NC_029701	*U. linza*	22	TTCTAATAACTAGTGAACAAGT	2
	KU161104	*U. prolifera*	22	TTCTAATAACTAGTGAACAAGT	2
	NC_028538	*U. prolifera*	22	TTCTAATAACTAGTGAACAAGT	2
**In chloroplast genome**					
4 groups	KX595276	*U. compressa*	6	AATATA	4
	W-178	*U. mutabilis*	6	AATATA	4
	KX595276	*U. compressa*	11	TTTAATTATTA	3
	W-178	*U. mutabilis*	11	TTTAATTATTA	3
	KX595276	*U. compressa*	12	AATTTAAAACAA	2
	W-178	*U. mutabilis*	12	AATTTAAAACAA	2
	KX595276	*U. compressa*	13	TTTATTATATTAA	2
	W-178	*U. mutabilis*	13	TTTATTATATTAA	2
2 groups	NC_036137	*U. prolifera*	6	AAGCTC	4
	NC_030312	*U. linza*	6	AAGCTC	9
	NC_036137	*U. prolifera*	15	AATGATTCAAATAAA	2
	NC_030312	*U. linza*	15	AATGATTCAAATAAA	2

Fig. S1. Phylogenetic analysis of strains used for chloroplast genome sequencing in this study (SP1, SP2, JP1, JF2, JF3, JF11)and those from NCBI database according to *tuf*A (Tamura 3-parameter+Gmodel)(A) and ITS(Kimura 2-parameter model) (B) using the maximum likelihood method. SP1, SP2, JP1, JF2, JF3, and JF11 are the progeny of the *U. compressa* strain for sequencing the chloroplast genomes in this study. **(NCBI/GenBank accession numbers are given.)**

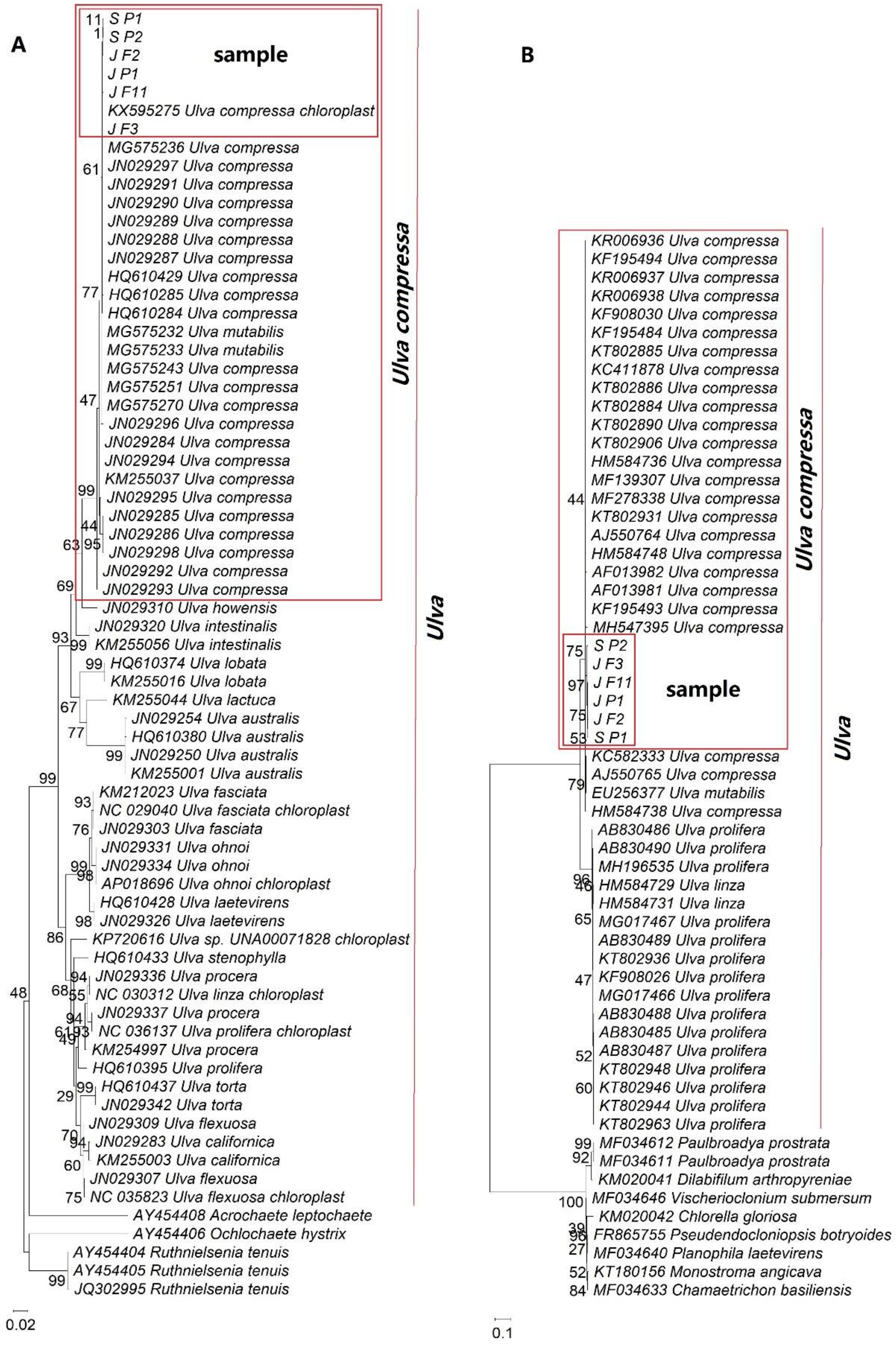

Fig. S2. Phylogenetic analysis using coding genes in chloroplast genomesin the genus *Ulva* by the maximum likelihood method. Each of the individual gene phylogenetic analysis was consistent with the tree obtained from whole chloroplast genomes. These genes and related tree models were as follows:
***tuf*A, *ycf*20**:Tamura 3-parameter+I model; ***atp*B, *cem*A, *chl*I, *clp*P, *pet*A, *rpl*5**: Tamura 3-parameter+G+I model; ***rbc*L, *rpo*A, *rpo*B, *rpo*C1**:General Time Reversible+G model; ***fts*H, *rpo*C2**:General Time Reversible+G+I model; ***psa*A**: Hasegawa-Kishino-Yano+G model; ***psb*I, *ycf*12**:Tamura 3-parameter model; ***pet*B, *rps*11, *rps*7, *ycf*4**: Tamura 3-parameter+G model. **(NCBI/GenBank accession numbers are given.)**

***tuf*A**

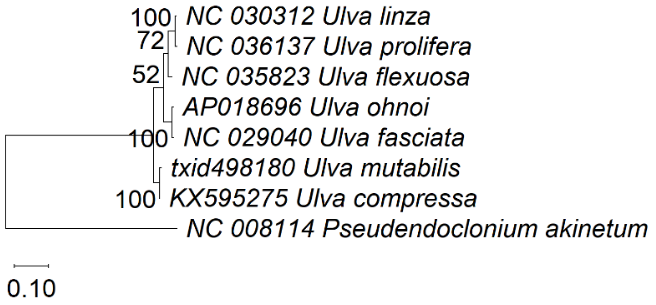

***ycf*20**

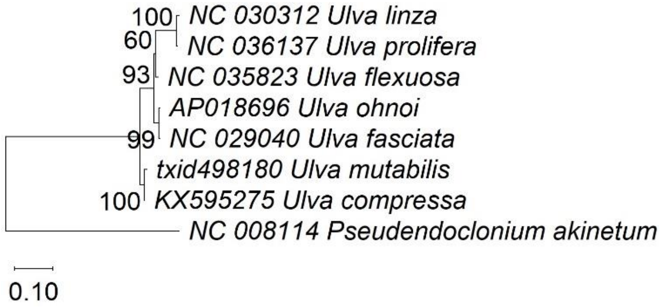

***atp*B**

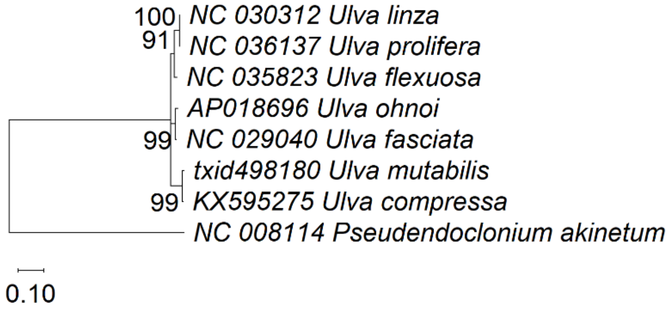

_cem_A

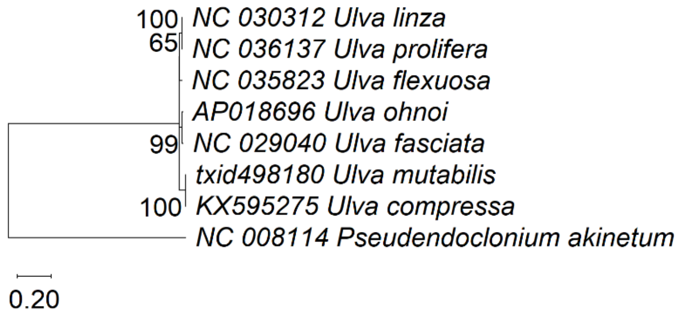

_chl_I

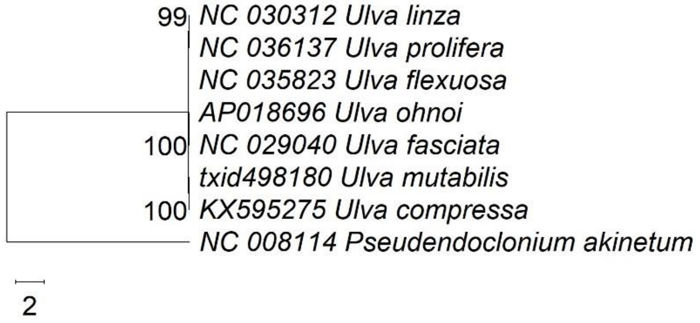

_clp_P

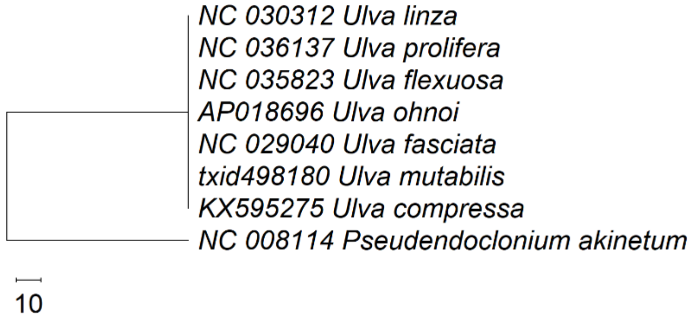

_pet_A

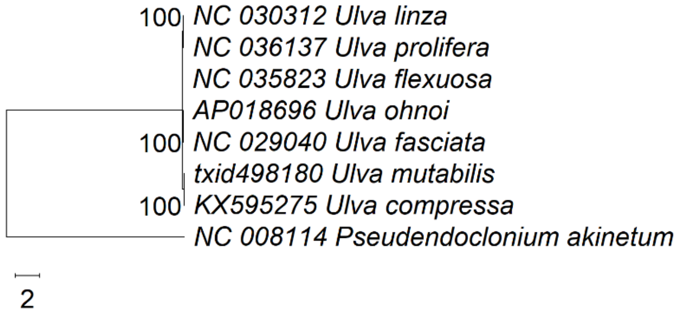

***rpl*5**

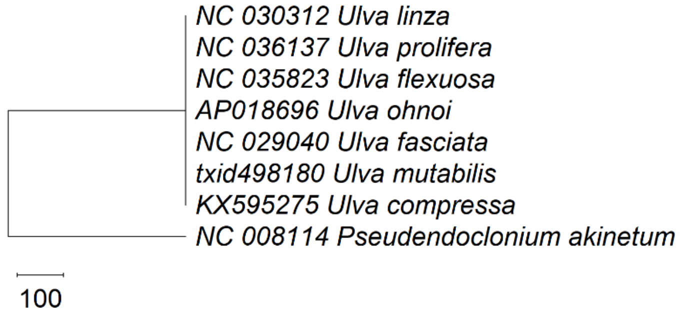

***rbc*L**

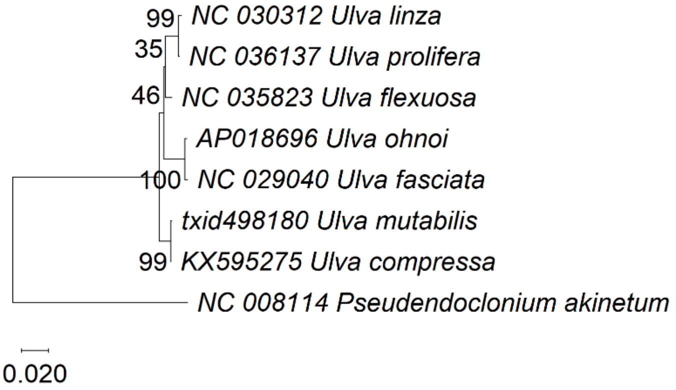

***rpo*A**

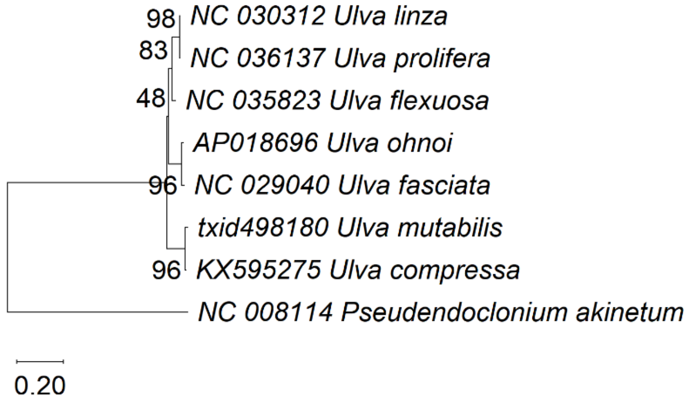

***rpo*B**

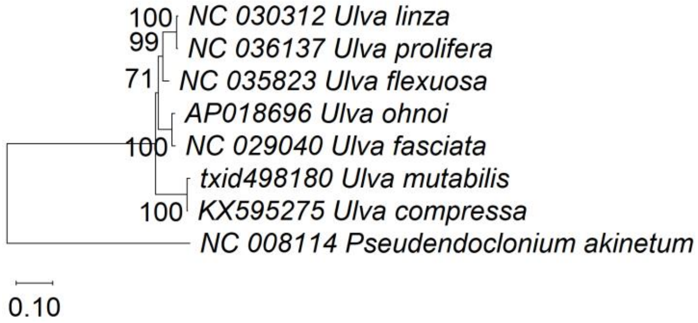

***rpo*C1**

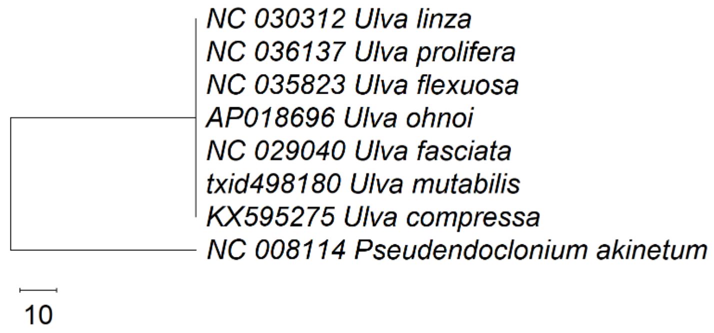

***fts*H**

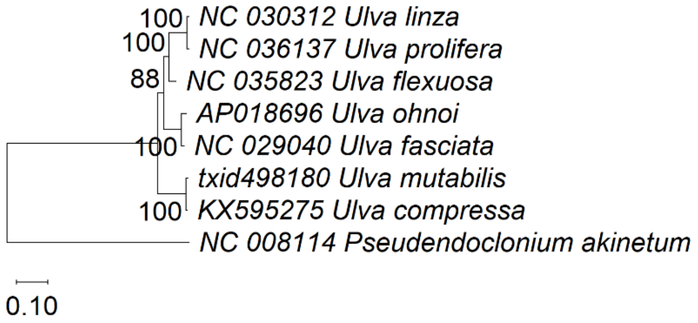

***rpo*C2**

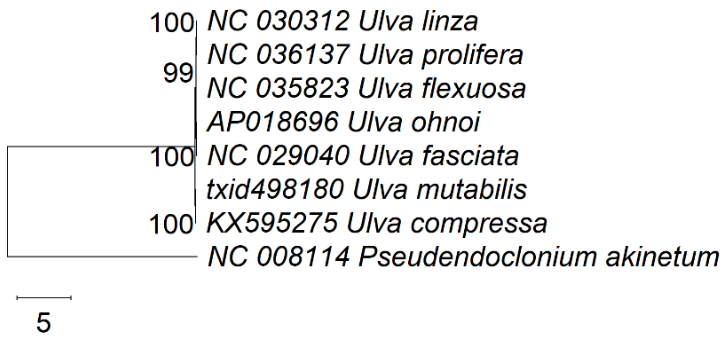

***psa*A**

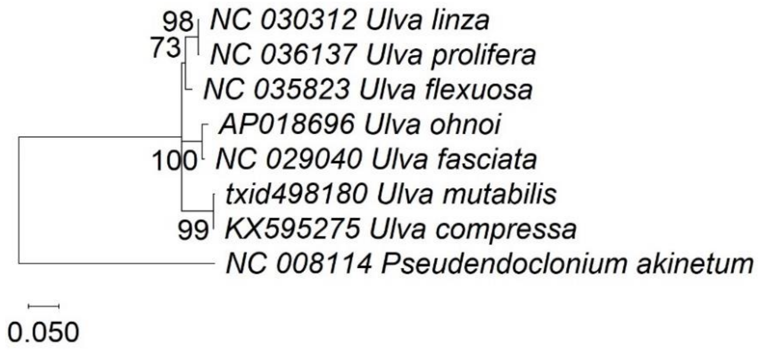

***psb*I**

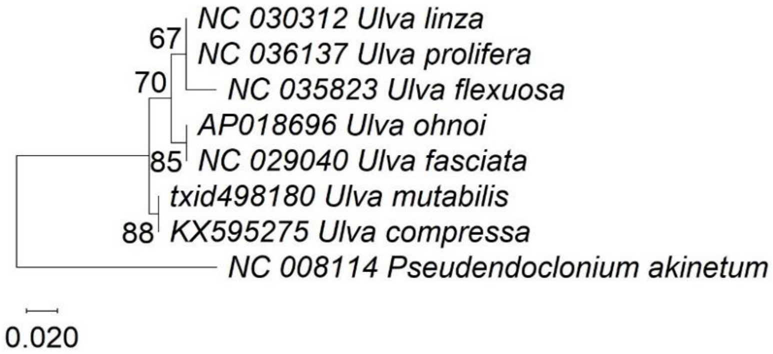

***ycf*12**

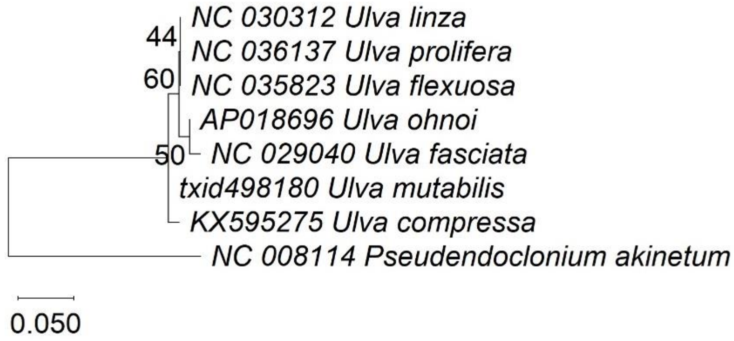

***pet*B**

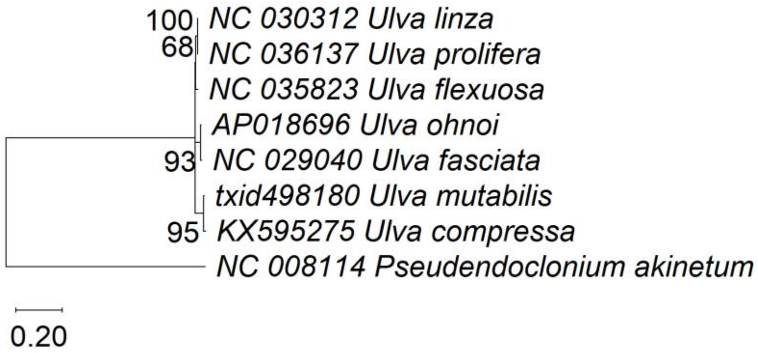

***rps*11**

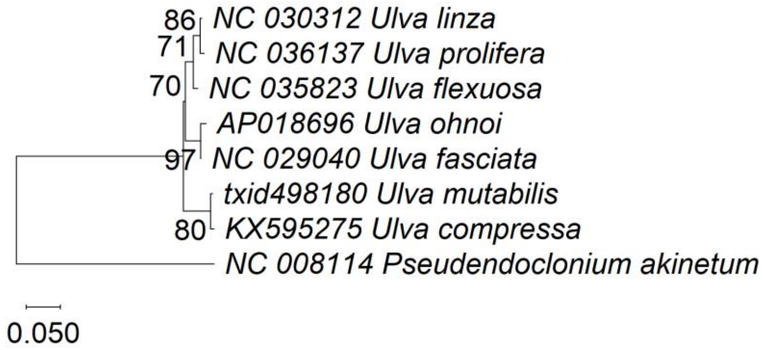

***rps*7**

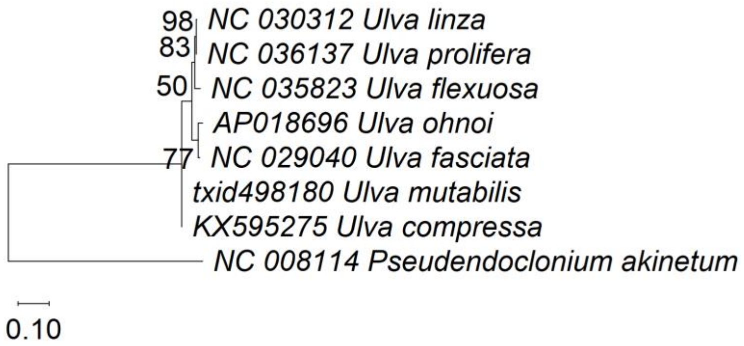
98
83
50
77
NC 030312 Ulva linza
NC 036137 Ulva prolifera
NC 035823 Ulva flexuosa
AP018696 Ulva ohnoi
NC 029040 Ulva fasciata
txid498180 Ulva mutabilis
KX595275 Ulva compressa
NC 008114 Pseudendoclonium akinetum
0.10

***ycf*4**

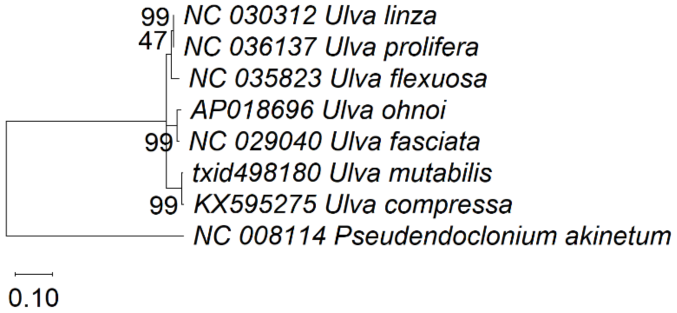
99
47
99
99
NC 030312 Ulva linza
NC 036137 Ulva prolifera
NC 035823 Ulva flexuosa
AP018696 Ulva ohnoi
NC 029040 Ulva fasciata
txid498180 Ulva mutabilis
KX595275 Ulva compressa
NC 008114 Pseudendoclonium akinetum
0.10

Fig. S3. Phylogenetic analysis of RNA sequences from the chloroplast genome of the genus *Ulva*, respectively, by the maximum likelihood method. A, *rrn*5, Tamura 3-parameter model; B, *rrn*16, Hasegawa-Kishino-Yano+G model; and C, *rrn*23, Tamura 3-parameter model. **(NCBI/GenBank accession numbers are given.)**

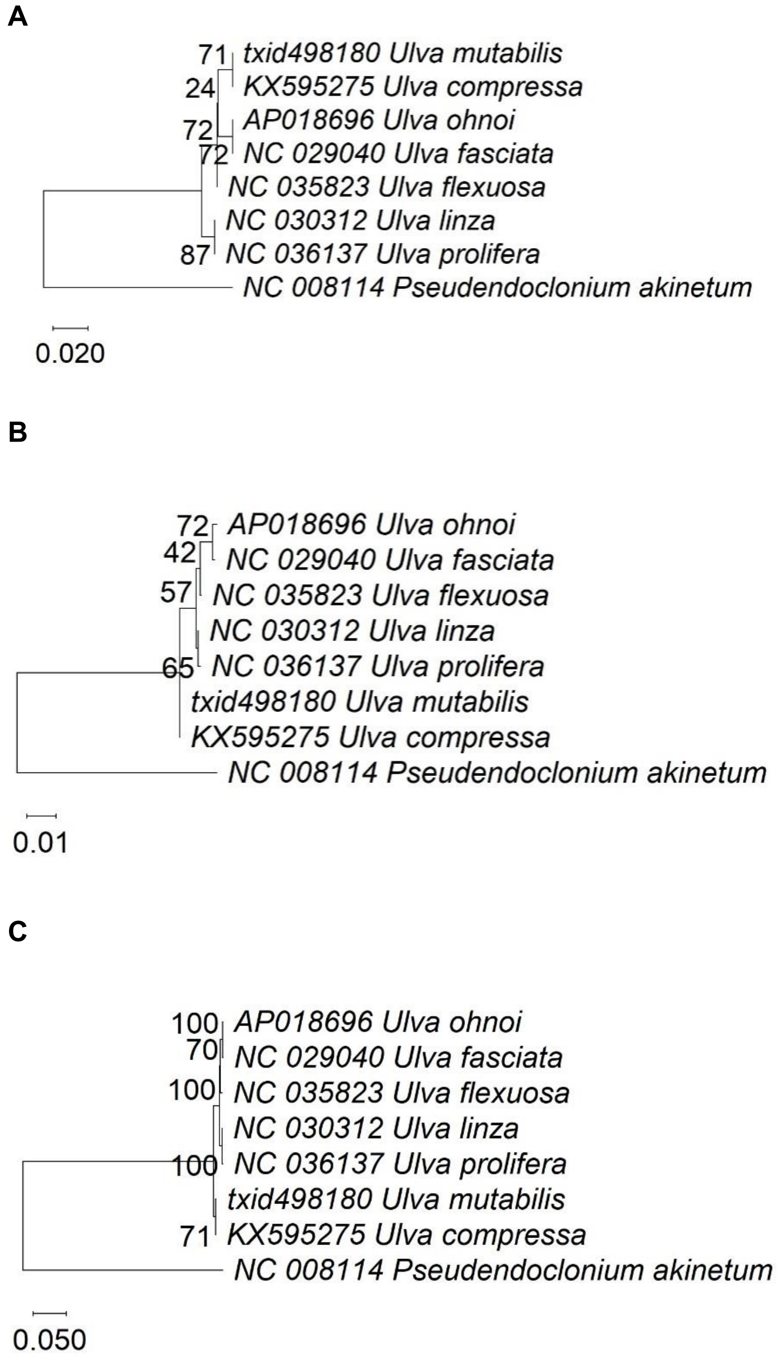

Fig. S4. Phylogenetic analysis of whole aligned *t*RNA sequence in the chloroplast genome of the genus *Ulva* by the maximum likelihood method in Kimura 2-parameter model. (NCBI/GenBank accession numbers are given.)

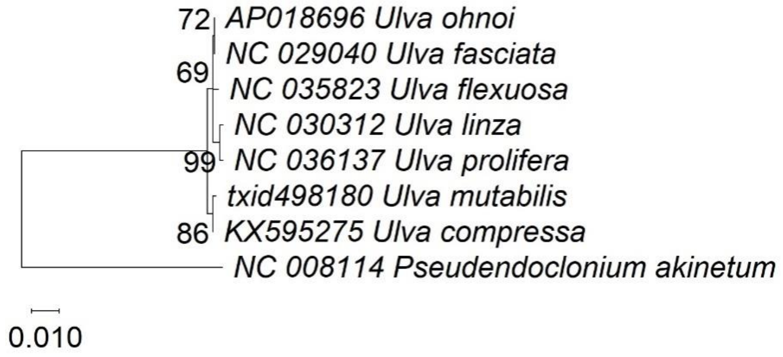

Fig. S5. Phylogenetic analysis of *U. ohnoi* and those from the NCBI database according to *tuf*A using the maximum likelihood method in Tamura 3-parameter model. Sample "*Ohnoi* in this study" represented the *U. ohnoi* strain used for PCR followed by sequencing in this study. **(NCBI/GenBank accession numbers are given.)**

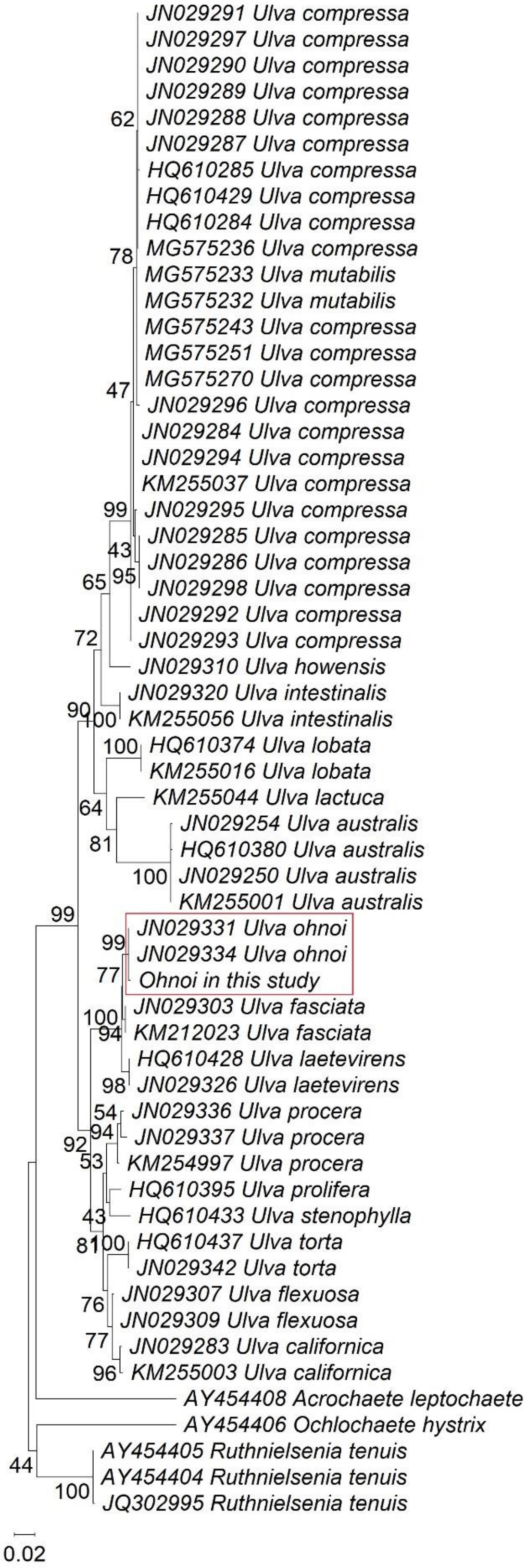

Supplement: S1 File — (PDF) [file pone.0250968.s001.pdf]
