# Supplementary material for: Screening and verification of extranuclear genetic markers in green tide algae from the Yellow Sea
Source: PLoS One. 2021 Jun 1;16(6):e0250968. doi: 10.1371/journal.pone.0250968 (PMC8168861; doi:10.1371/journal.pone.0250968)
Supplement: S2 File — (PDF) [file pone.0250968.s002.pdf]

All original figures for

Screening and verification of extranuclear genetic markers in green tide algae from the Yellow Sea

Chuner Cai[1,2*], Kai Gu[1], Hui Zhao[1], Sophie Steinhagen[3],

Peimin He[1], Thomas Wichard[2*]

[1] College of Marine Ecology and Environment, Shanghai Ocean University, Shanghai, China

[2] Institute for Inorganic and Analytical Chemistry, Jena School for Microbial Communication, Friedrich Schiller University Jena, Jena, Germany

[3] Department of Marine Sciences-Tjärnö Marine Laboratory, University of Gothenburg, Strömstad, Sweden

*** Corresponding authors**

E-mail

Chuner Cai: cecai@shou.edu.cn

Thomas Wichard: thomas.wichard@uni-jena.de

The following images of the gel electrophoresis were used for the presentation of the results in Figure 6, 7 and 8.

Figures 6

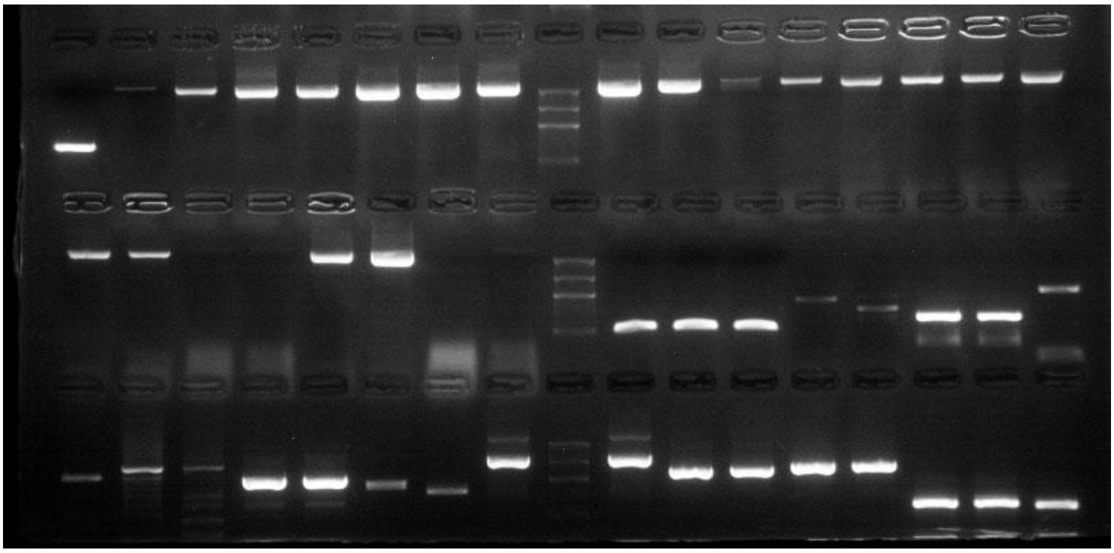

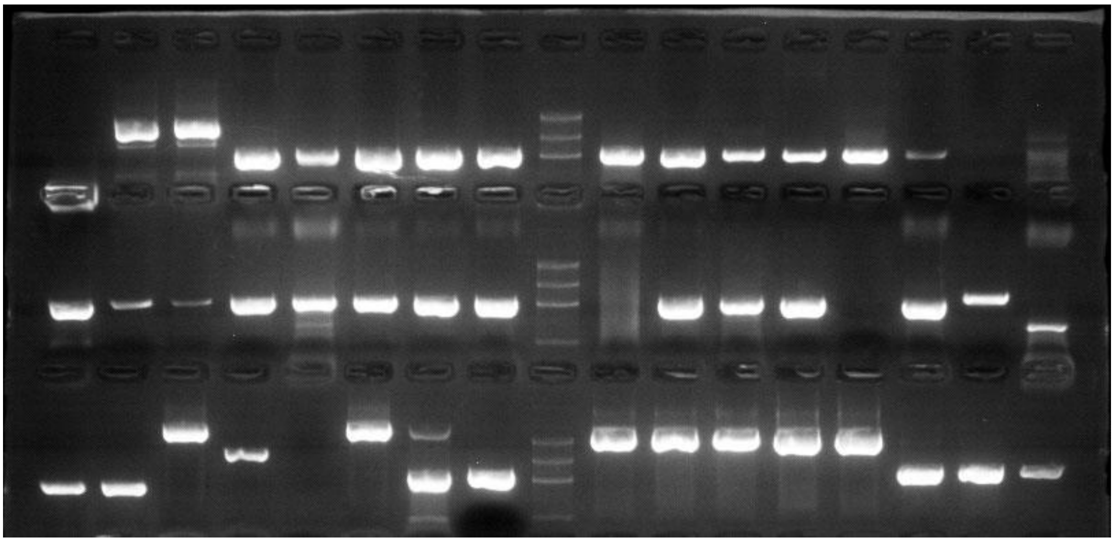

Figures 7A

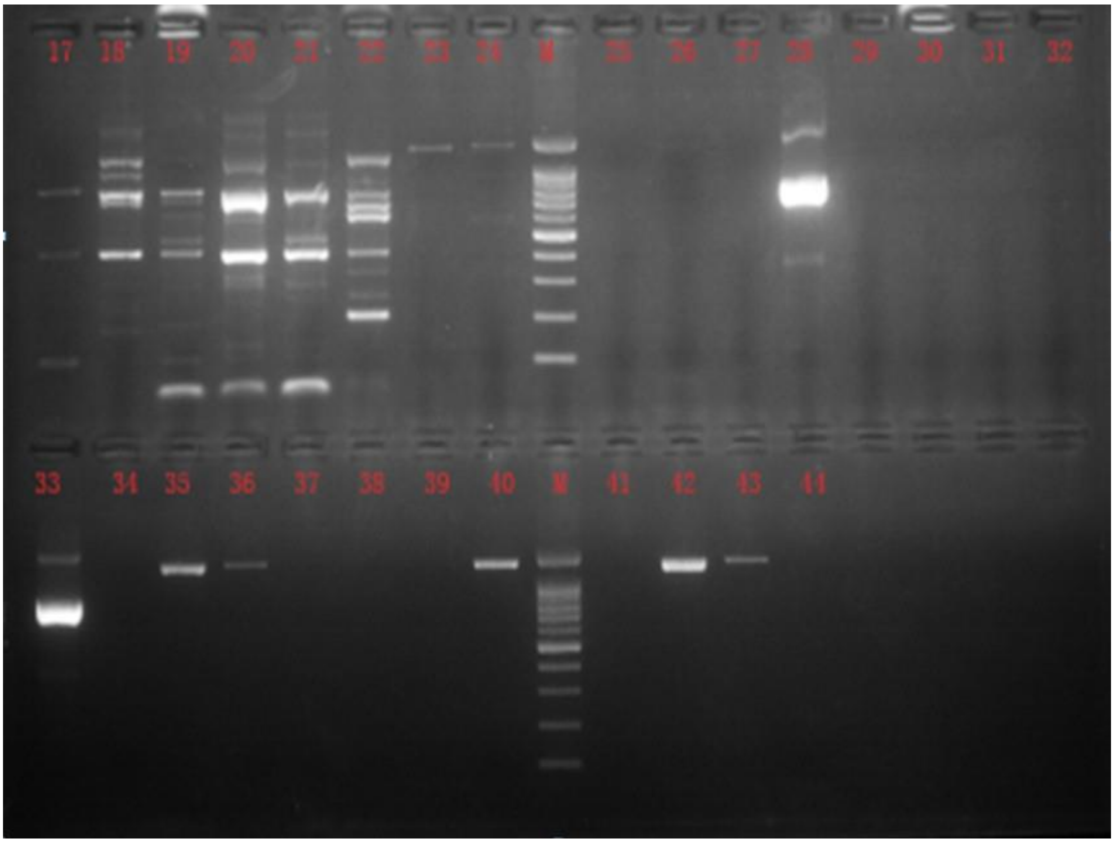
17 18 19 20 21 22 23 24 M 25 26 27 28 29 30 31 32
33 34 35 36 37 38 39 40 M 41 42 43 44

Figures 7B

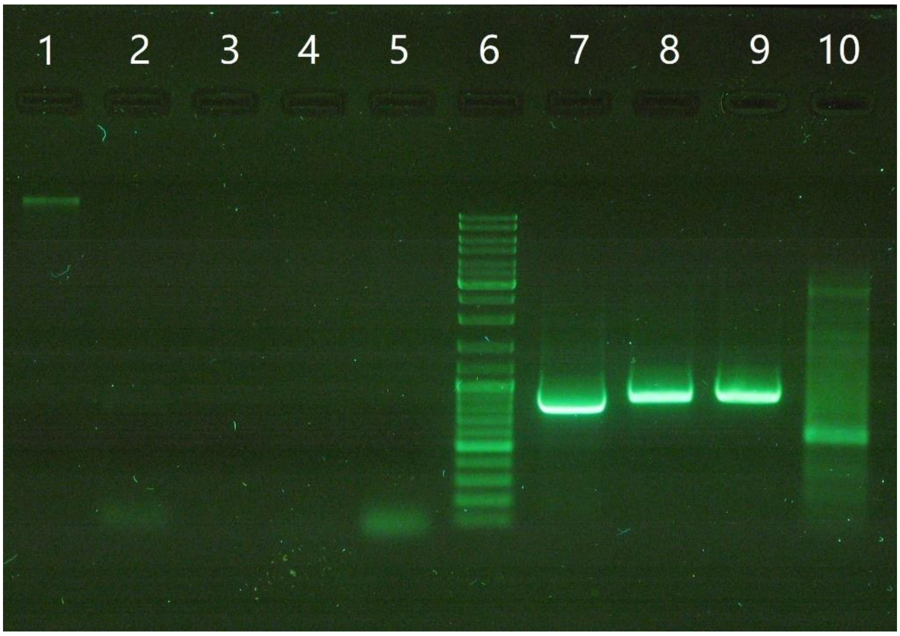

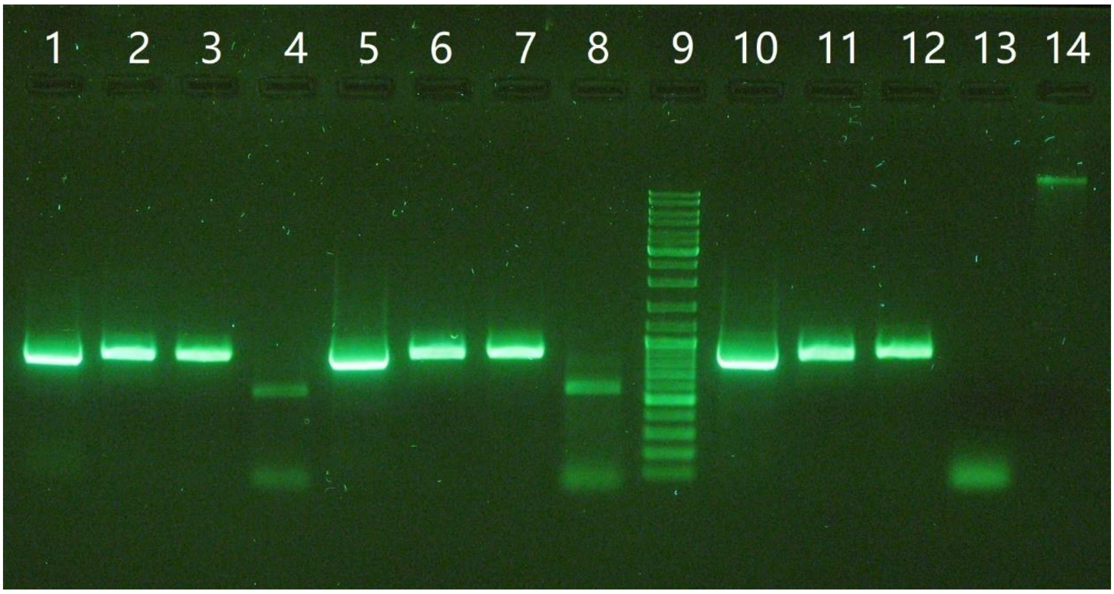

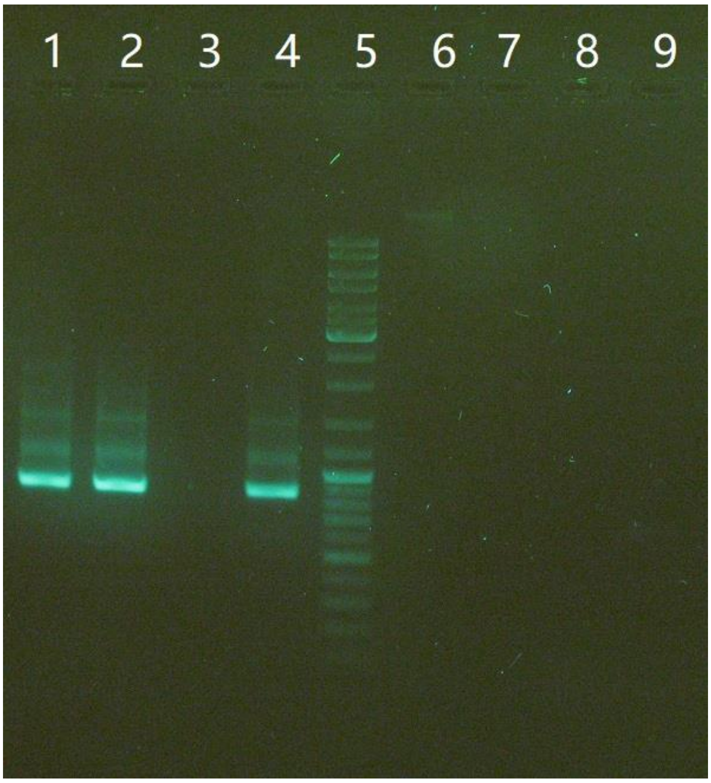
1 2 3 4 5 6 7 8 9

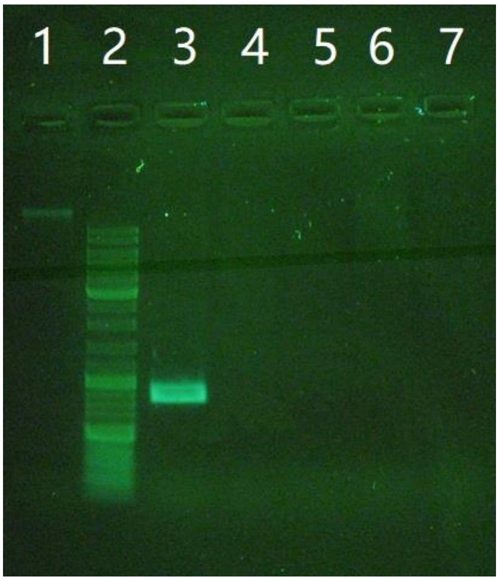
1 2 3 4 5 6 7

Figures 8

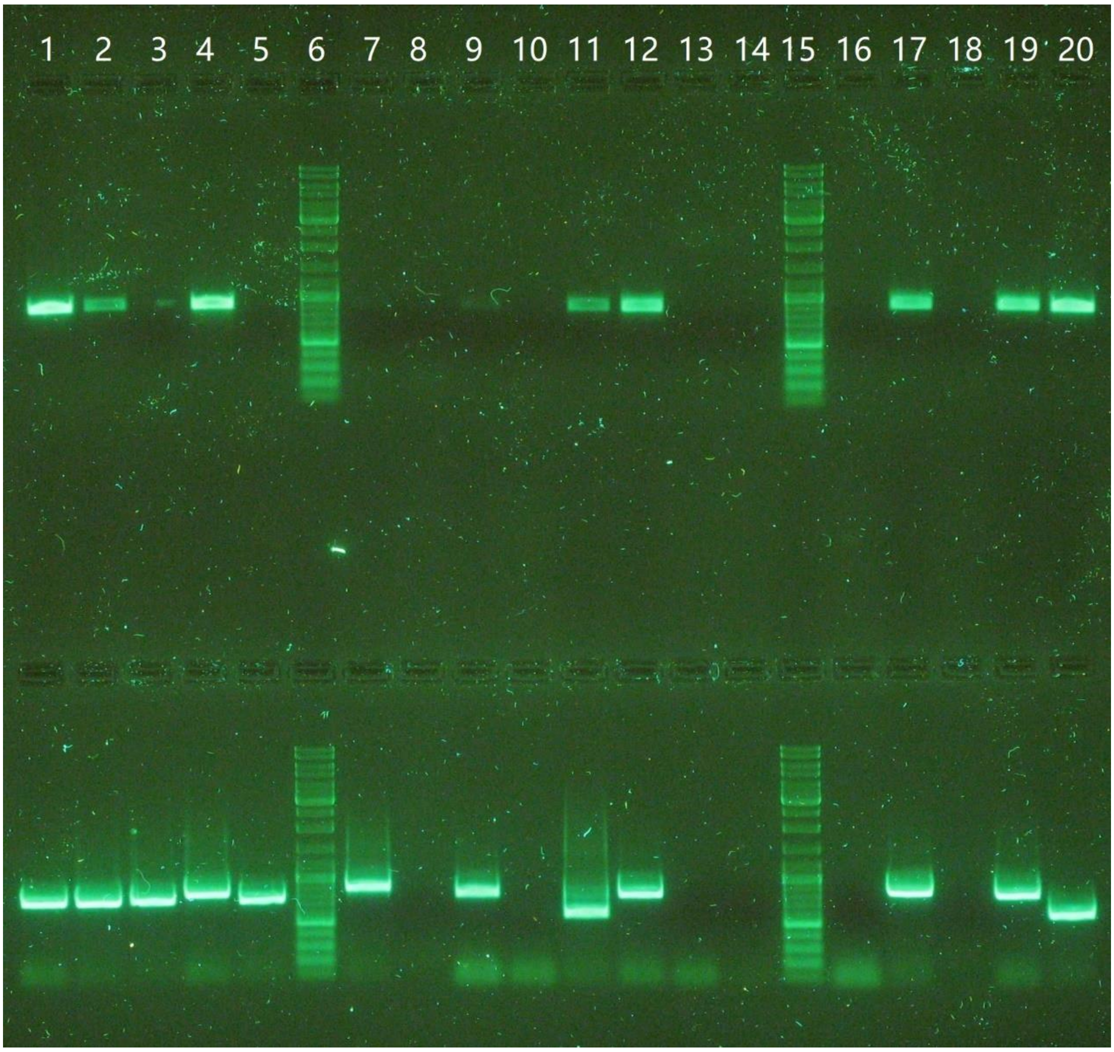

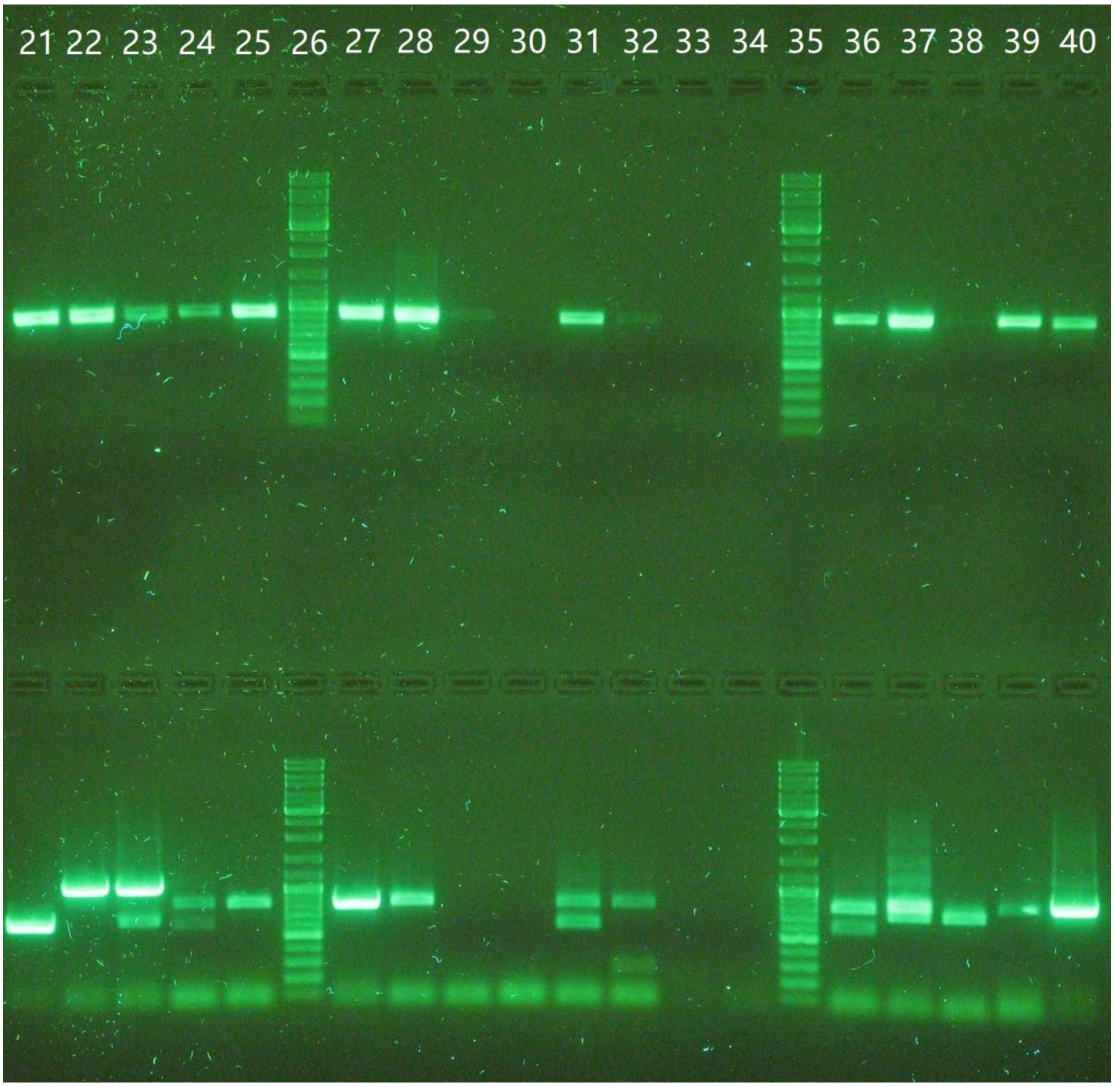
21 22 23 24 25 26 27 28 29 30 31 32 33 34 35 36 37 38 39 40

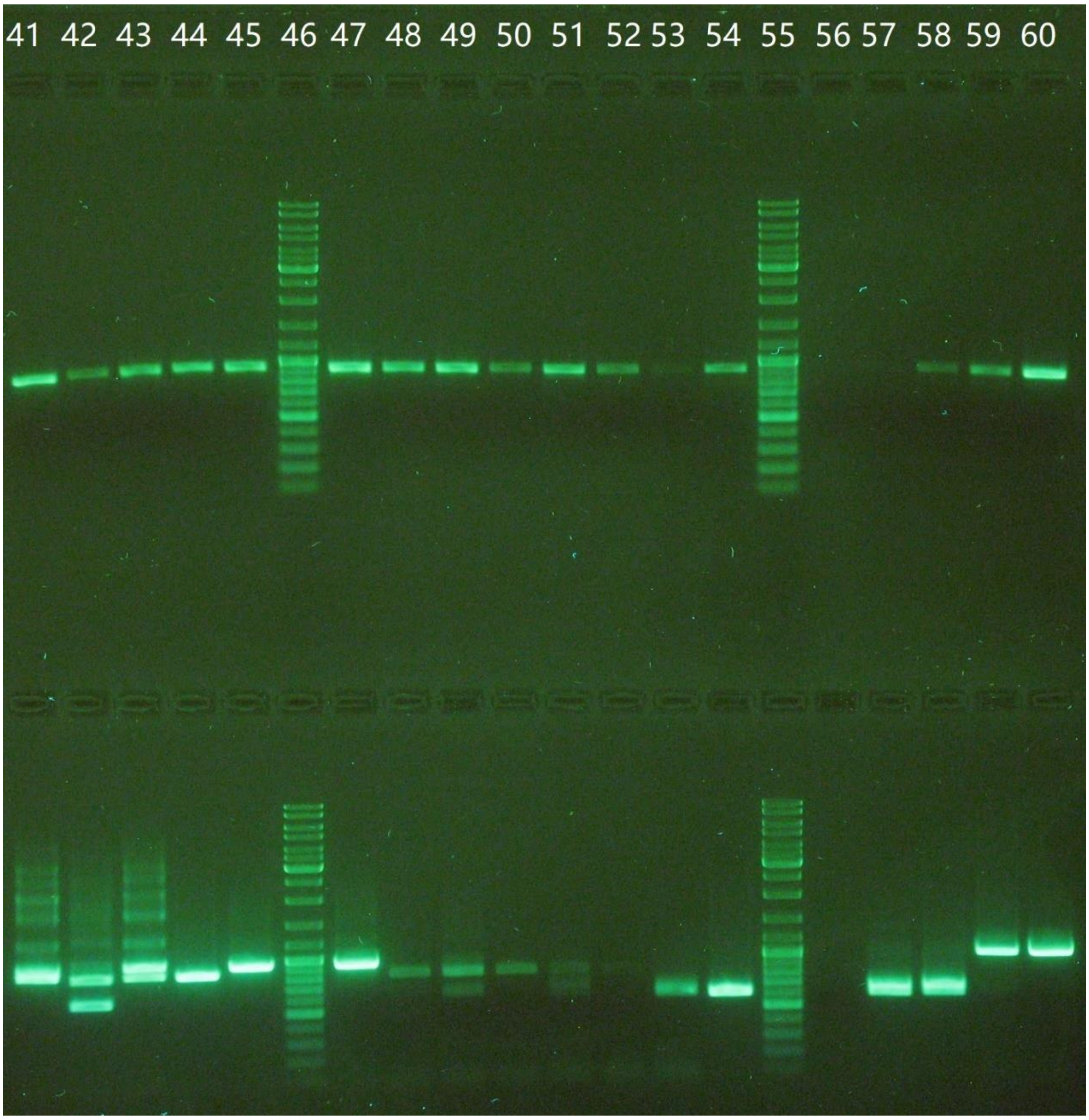
41 42 43 44 45 46 47 48 49 50 51 52 53 54 55 56 57 58 59 60

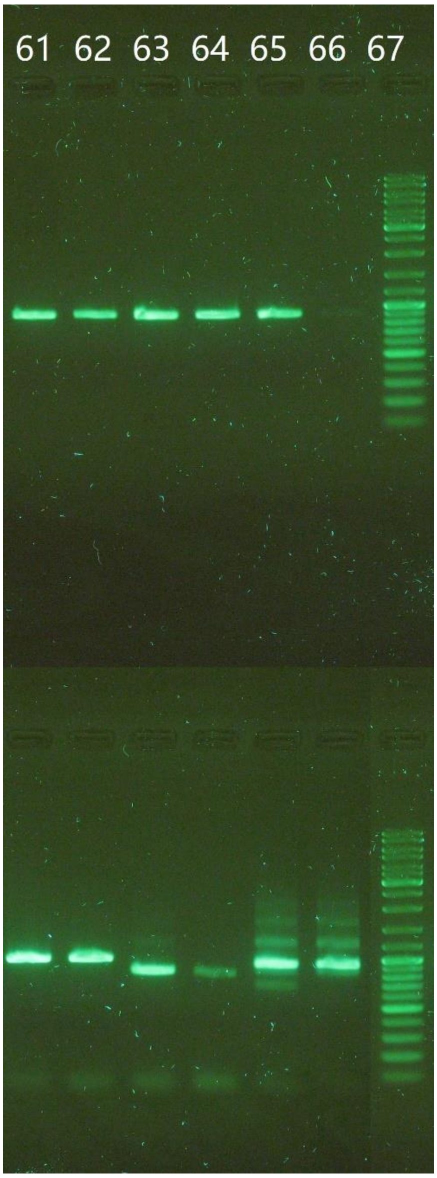
61 62 63 64 65 66 67
